# Protein aggregation and calcium dysregulation are hallmarks of familial Parkinson’s disease in midbrain dopaminergic neurons

**DOI:** 10.1038/s41531-022-00423-7

**Published:** 2022-11-24

**Authors:** Gurvir S. Virdi, Minee L. Choi, James R. Evans, Zhi Yao, Dilan Athauda, Stephanie Strohbuecker, Raja S. Nirujogi, Anna I. Wernick, Noelia Pelegrina-Hidalgo, Craig Leighton, Rebecca S. Saleeb, Olga Kopach, Haya Alrashidi, Daniela Melandri, Jimena Perez-Lloret, Plamena R. Angelova, Sergiy Sylantyev, Simon Eaton, Simon Heales, Dmitri A. Rusakov, Dario R. Alessi, Tilo Kunath, Mathew H. Horrocks, Andrey Y. Abramov, Rickie Patani, Sonia Gandhi

**Affiliations:** 1 The Francis Crick Institute, 1 Midland Road, London, NW1 1AT UK; 2 Department of Clinical and Movement Neurosciences, UCL Queen Square Institute of Neurology, Queen Square, London, WC1N 3BG UK; 3 Aligning Science Across Parkinson’s (ASAP) Collaborative Research Network, Chevy Chase, MD 20815 USA; 4 Medical Research Council (MRC) Protein Phosphorylation and Ubiquitylation Unit, School of Life Sciences, University of Dundee, Dow Street, Dundee, DD1 5EH UK; 5 EaStCHEM School of Chemistry, University of Edinburgh, Edinburgh, EH9 3FJ UK; 6 Center for Regenerative Medicine, University of Edinburgh, Edinburgh, EH16 4UU UK; 7 Department of Clinical and Experimental Epilepsy, UCL Queen Square Institute of Neurology, London, WC1N 3BG UK; 8 UCL Great Ormond Street Institute of Child Health, London, WC1N 1EH UK; 9 Department of Neurodegenerative Diseases, UCL Queen Square Institute of Neurology, Queen Square, London, WC1N 3BG UK; 10 Rowett Institute, University of Aberdeen, Ashgrove Rd West, Aberdeen, AB25 2ZD UK; 11 Department of Neuromuscular Disease, UCL Queen Square Institute of Neurology, Queen Square, London, WC1N 3BG UK

**Keywords:** Cellular neuroscience, Stem-cell differentiation, Induced pluripotent stem cells

## Abstract

Mutations in the *SNCA* gene cause autosomal dominant Parkinson’s disease (PD), with loss of dopaminergic neurons in the substantia nigra, and aggregation of α-synuclein. The sequence of molecular events that proceed from an *SNCA* mutation during development, to end-stage pathology is unknown. Utilising human-induced pluripotent stem cells (hiPSCs), we resolved the temporal sequence of *SNCA-*induced pathophysiological events in order to discover early, and likely causative, events. Our small molecule-based protocol generates highly enriched midbrain dopaminergic (mDA) neurons: molecular identity was confirmed using single-cell RNA sequencing and proteomics, and functional identity was established through dopamine synthesis, and measures of electrophysiological activity. At the earliest stage of differentiation, prior to maturation to mDA neurons, we demonstrate the formation of small β-sheet-rich oligomeric aggregates, in *SNCA*-mutant cultures. Aggregation persists and progresses, ultimately resulting in the accumulation of phosphorylated α-synuclein aggregates. Impaired intracellular calcium signalling, increased basal calcium, and impairments in mitochondrial calcium handling occurred early at day 34–41 post differentiation. Once midbrain identity fully developed, at day 48–62 post differentiation, *SNCA*-mutant neurons exhibited mitochondrial dysfunction, oxidative stress, lysosomal swelling and increased autophagy. Ultimately these multiple cellular stresses lead to abnormal excitability, altered neuronal activity, and cell death. Our differentiation paradigm generates an efficient model for studying disease mechanisms in PD and highlights that protein misfolding to generate intraneuronal oligomers is one of the earliest critical events driving disease in human neurons, rather than a late-stage hallmark of the disease.

## Introduction

Parkinson’s disease (PD) is a progressive neurodegenerative disease primarily characterised by the loss of dopaminergic neurons in the substantia nigra of the midbrain^1^. Studying the mechanisms that contribute to disease is of immense importance, but has been somewhat limited by a paucity of relevant human models. The development of human-induced pluripotent stem cells (hiPSCs) from patients with familial forms of PD has vastly improved our capability to model and study PD. Taking this advance further, directed differentiation strategies to generate the relevant cell type from the relevant brain region, should allow us to discover mechanisms of disease and define the basis for selective cellular vulnerability. For PD, amongst the earliest and most vulnerable cell populations is the midbrain dopaminergic neuron, and therefore modelling disease with precision requires the ability to robustly generate this neuronal type.

Several protocols have transformed our ability to generate midbrain dopaminergic (mDA) neurons^2–5^: these mimic in vivo developmental programmes, directing neural induction via dual-SMAD inhibition^6^, and subsequent patterning via activation of sonic hedgehog (Shh), Wnt, and fibroblast growth factor 8 (FGF8) signalling through the use of small-molecules and/or recombinant morphogens. The resulting cells acquire midbrain-specific molecular attributes, based on the expression of key genes and proteins, as well as functional and physiological characteristics resembling in vivo mDA neurons^7^. The efficiency of mDA neuron production varies between current differentiation protocols^8^, and cellular heterogeneity can be challenging when modelling disease. Against this background, we adapted well-established protocols to obtain highly enriched mDA neurons, and we tested their capacity to reliably model key aspects of PD pathogenesis and specifically to identify early phenotypes.

The pathological hallmark of PD is the presence of insoluble aggregated forms of the protein α -synuclein, encoded by the *SNCA* gene^9^. Modelling synucleinopathy in vitro may therefore be achieved by utilising hiPSC lines with *SNCA* mutations. A range of mutations and multiplications have been discovered in *SNCA*, including the p.A53T point mutation and the triplication of the *SNCA* locus. Both of these genetic changes cause the early onset and rapidly progressive form of PD^10–12^. HiPSC-derived models harbouring *SNCA* mutations have highlighted a range of cellular phenotypes that may lead to cell vulnerability, including mitochondrial dysfunction^13–15^, ER stress^16^, lysosomal dysfunction^17^, oxidative stress, and calcium dysregulation^18,19^. Nonetheless, disease models are dominated by multiple forms of cellular stress, and it has not been possible to determine the emergence of cellular dysfunction, and resolve the events spatially and temporally.

In this study, we established a modified approach to obtain highly enriched mDA neurons and we demonstrate their capacity to faithfully model key aspects of PD pathogenesis with temporal resolution. Our study reveals that protein misfolding to generate intraneuronal oligomers, and impaired calcium signalling, are early events in patient-derived PD neurons, and precede the appearance of mitochondrial, oxidative and lysosomal pathology.

## Results

### Highly enriched midbrain dopaminergic neurogenesis from hiPSCs

**Fig. 1:**
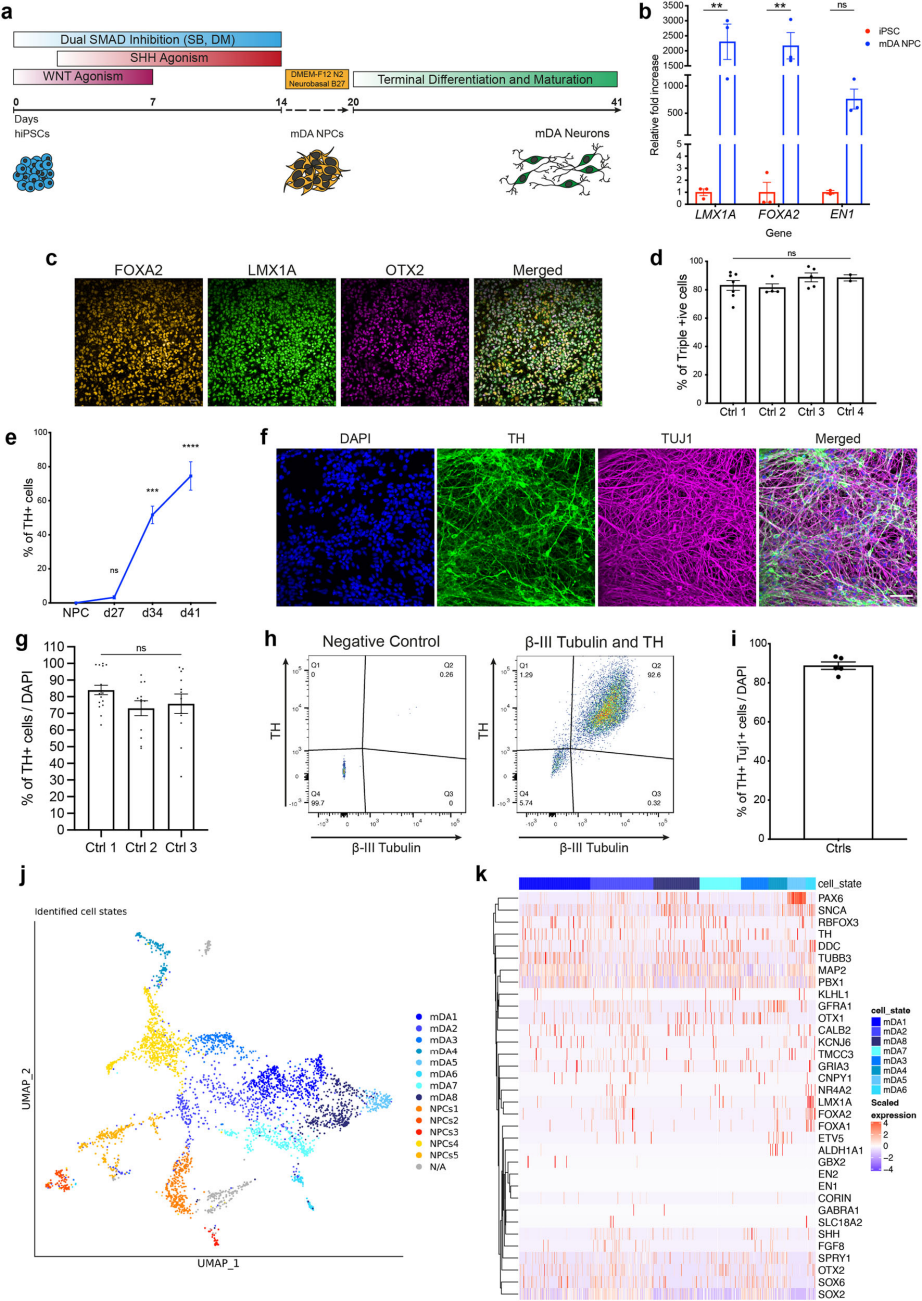
Characterisation of an enriched population of mDA NPCs and neurons. **a** Differentiation protocol to generate mDA neurons. **b** Quantitative PCR at day 14–20 of differentiation showing mRNA for *LMX1A*, *FOXA2*, and *EN1* relative to hiPSCs (ns *P* > 0.05, **P < 0.005). **c** Representative ICC images showing expression of FOXA2, LMX1A, and OTX2 (scale bar = 50 μm). **d** Quantification showing >80% of cells co-express FOXA2, LMX1A and OTX2 (ns *P* > 0.05, ordinary one-way ANOVA). **e** Quantification of ICC images showing the increase in expression of the mDA marker, TH over differentiation (weeks) (ns *P* > 0.05, ***P < 0.0005, ****P < 0.0001, ordinary one-way ANOVA). **f** Representative ICC images showing TH and TUJ1 expression after 41 days of differentiation (scale bar = 50 μm). **g** ICC Quantification showing approximately 80% cells express TH (ns *P* > 0.05, ordinary one-way ANOVA). **h** Representative dot plots of single-cell suspensions showing % TH and β-III Tubulin +ve cells (day 41). A negative control (DAPI only) was used to determine quantification thresholds (*n* = 10,000 events recorded per measurement). **i** Quantification of flow cytometry showing >80% of DAPI-positive cells co-express TH and β-III Tubulin. **j** A UMAP plot showing the 14 clusters identified from single-cell RNA-seq after day 48 of differentiation. Neuronal mDA (mDA1–8) clusters are in blue, and NPC clusters (NPCs1–5) are in red, orange and yellow. An unidentified cluster (N/A) is coloured in grey. **k** Heatmap showing expression of genes in clusters identified as mDA neurons (mDA1–8). Each line represents a cell from that cluster. All data plotted as ±s.e.m. All *N* numbers for each experiment can be found in Supplementary Table 5.

To generate mDA neurons from hiPSCs, neural conversion was achieved using dual-SMAD inhibition^6^ followed by midbrain patterning using small molecule agonists of Shh and Wnt, purmorphamine and CHIR99021, respectively (Fig. 1a). After 14–20 days, we confirmed mDA neural precursor cell (NPC) identity using RT-PCR for *LMX1A, FOXA2*, and *EN1* (*P* < 0.005) (Fig. 1b) and immunocytochemistry (ICC) for LMX1A, FOXA2 and OTX2 (Fig. 1c). Crucially, high co-expression of all three markers, LMX1A, FOXA2 and OTX2, confirmed an enriched culture of mDA NPCs (Ctrl 1: 83% ± 3.5, Ctrl 2: 81.4% ± 2.4, Ctrl 3: 88.7% ± 3.1, Ctrl 4: 88.4% ± 2.2) (Fig. 1d). After midbrain patterning, NPCs were maintained in culture for 4 days before terminal differentiation. Neuronal differentiation was induced using a Notch pathway inhibitor and a Rho-associated protein kinase (ROCK) inhibitor, encouraging cell cycle exit and cell survival, respectively. Maturation of terminally differentiated mDA neurons was confirmed by an increase of TH-positive cells (Fig. 1e and Supplementary Fig. 1a). Over 75% of TH-positive cells were obtained after 41 days of differentiation using ICC (Ctrl 1: 84.1% ± 2.8, Ctrl 2: 73.1% ± 4.5, Ctrl 3: 75.8% ± 5.8) (Fig. 1f, g), across three different hiPSC lines and four neuronal inductions (Supplementary Fig. 1b). Flow cytometry confirmed ~88% expression of both TH and β-III Tubulin (88.8% ± 1.9) (Fig. 1h, i and Supplementary Fig. 1c, d). Overall, the data confirm the high efficiency of neuronal differentiation, specifically into TH-positive neurons in 80–90% of the whole culture, with comparable efficiencies across three control hiPSC lines. qPCR for specific genes, including *NR4A2* (Nurr1*)*, *KCNJ6* (GIRK2), and *SLC6A3* (DAT) at day 41–48-old mDA neurons showed an increase in the expression of these transcriptional markers of terminally differentiated mDA neurons relative to midbrain NPCs (Supplementary Fig. 1e). ICC quantification using a knockout validated GIRK2 antibody confirmed the expression of GIRK2 in mDA neurons which further supports the midbrain identity (Supplementary Fig. 1f, g), given that GIRK2 is highly expressed in dopaminergic neurons in the A9 region of the midbrain.

**Fig. 2:**
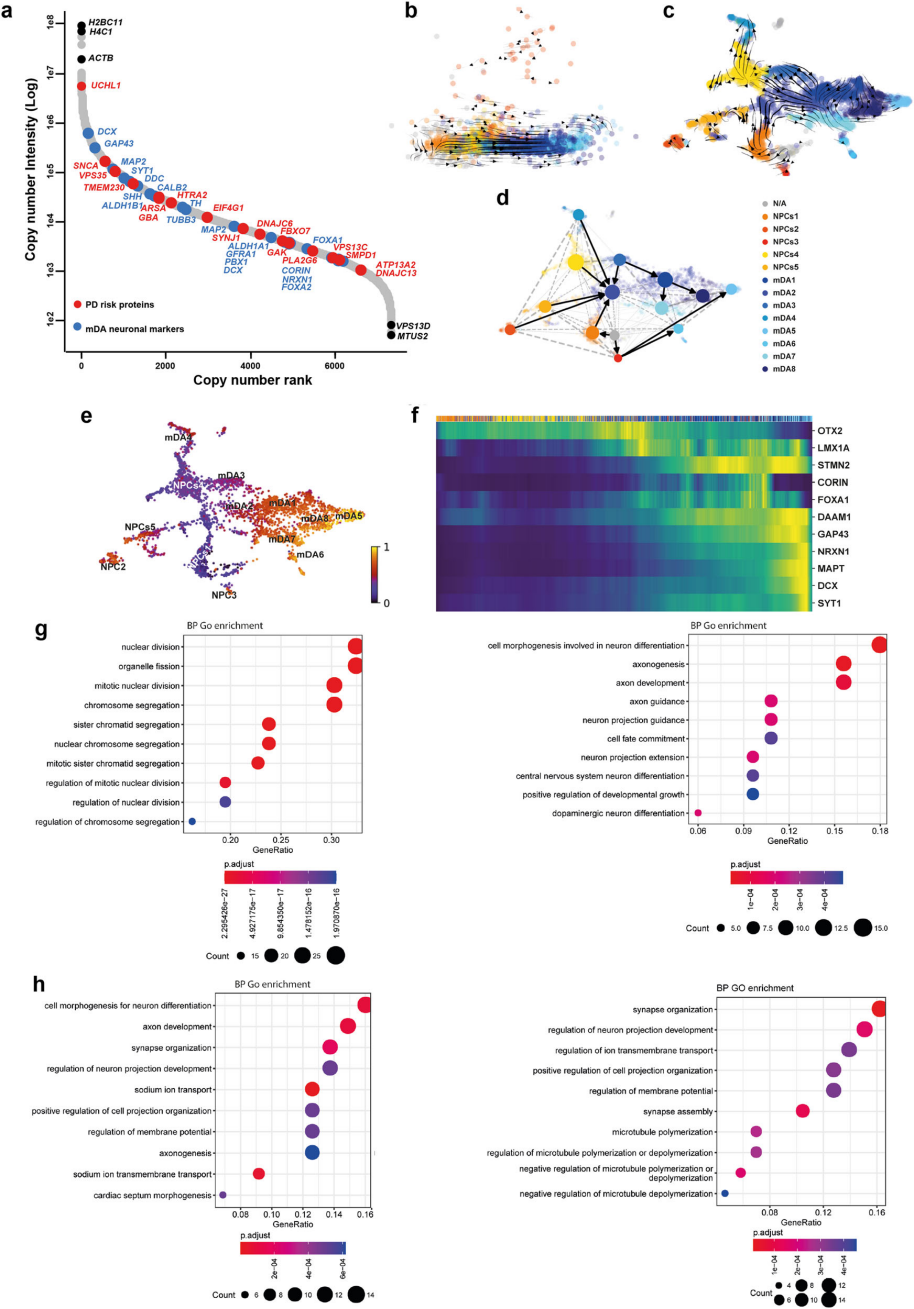
RNA velocity demonstrates developmental streams from NPCs into mDA neurons reminiscent of a developing midbrain. **a** Rank abundance plot showing copy number of proteins from proteomic analysis with expression of mDA proteins in blue and PD-linked proteins in red. **b** PCA visualises the progression from precursor cells (NPCs) (yellow-red colours) towards mDA neuronal cells (blue colours). RNA velocity trajectories are indicated by arrows. **c** Velocity stream visualised in UMAP reveals a more detailed progression of the direction of differentiation. **d** PAGA graph showing the NPC cluster transition into mDA neuron clusters. **e** Velocity inferred latent time analysis visualised in UMAP, showing mDA clusters are later (brighter colour) in time than NPC clusters (darker colours). **f** A heatmap listing a selection of cluster driver genes sorted according to their inferred latent time. **g**, **h** GO enrichment analysis to clarify gene categories per cell cluster based on the functional characteristics of the driver genes. **g** Example of GO enrichment of two NPC clusters showing that some clusters are more proliferative (left graph), and some are more differentiated and on the neuronal pathway (right graph). **h** Example of two mDA neuron clusters showing complex neuronal pathways which are highly activated.

Single-cell RNA-seq was performed on day 48 mDA neurons. Cells were clustered into identities based on their transcriptional profiles, revealing 14 clusters, of which 8 expressed key mDA neuron genes, confirming their identity as mDA neurons (Fig. 1j). Key mDA genes expressed include GDNF receptor (*GFRA1*), *SOX6, PBX1, TH, KCNJ6*, *NR4A2*, and *DDC*, suggesting that the majority of captured cells were of mDA identity, and were abundant within the culture (Fig. 1k) (Supplementary Fig. 2a). Several clusters were similar to mDA NPCs, or early neurons (5 clusters) (Fig. 1j). These clusters expressed key NPC genes e.g *FOXA2, LMX1A, OTX2, FGF8, and SHH*, as well as mDA neuronal markers including *GFRA1*, *DDC*, *NR4A2*, *TH*, and *KCNJ6* (Supplementary Fig. 2b–d). In addition, most clusters expressed *SNCA*, highlighting the potential to use the protocol to model synucleinopathy (Supplementary Fig. 1h). Bulk proteomics was performed on terminally differentiated mDA neurons from three control lines across multiple inductions. Copy number analysis confirms the expression of key markers, including TH, DDC, FOXA2, and SHH (Fig. 2a), which varies across inductions and lines, but with no significant differences (Supplementary Fig. 2e). Copy number proteomics further confirmed the expression of several key proteins involved in PD in terminally differentiated mDA neurons (Fig. 2a).

We performed RNA velocity analysis, scVelo^20^ in single-cell RNA-seq data on day 48-old mDA neuronal cultures^21^. RNA velocity, i.e., the change in RNA abundance for each gene in a single cell, can be used to predict the future transcriptomic state of a cell, and thus infer developmental trajectories. The expected neuronal differentiation trajectory from the NPC clusters towards mature mDA neurons was confirmed (Fig. 2b, c), and additional differentiation trajectories from several mDA neuron clusters to other mDA neuron clusters were identified (Fig. 2d). The dynamical model used by scVelo allowed us to recover latent time, and reconstruct the temporal sequence of cellular fates: NPC cell clusters were earlier than mDA neurons in latent time, and were spread across latent time, suggesting different clusters were at different points on the path to form mature mDA neurons (Fig. 2e). The dynamic behaviour of the top genes for each cluster systematically detected by likelihoods (where the genes associated with mDA differentiation include *STMN2, DCX*, and *SYT1*, and the genes for NPC clusters include *OTX2, LMX1A* and *QKI*) can be seen across latent time (Fig. 2f) and across clusters (Supplementary Fig. 3b), as well as the different confidence probability of the predicted mDA neuronal cluster trajectory path (Supplementary Fig. 3c). We performed gene ontology (GO) enrichment^22^ for the top 100 identified genes for each cluster. NPC clusters were associated with GO terms related to proliferation, as well as neuronal differentiation (Fig. 2g). mDA neuron clusters showed enrichment in GO terms associated with complex synapse formation, as well as ion transport development (Fig. 2h) (Supplementary Table 4). Our analysis demonstrates that, at 48 days of differentiation, our method predominantly generates cells of mDA neuronal identity, with a clear developmental trajectory defined for the remaining NPCs to differentiate into mDA neurons.

### hiPSC-derived mDA neurons exhibit functional neuronal and dopaminergic identity

**Fig. 3:**
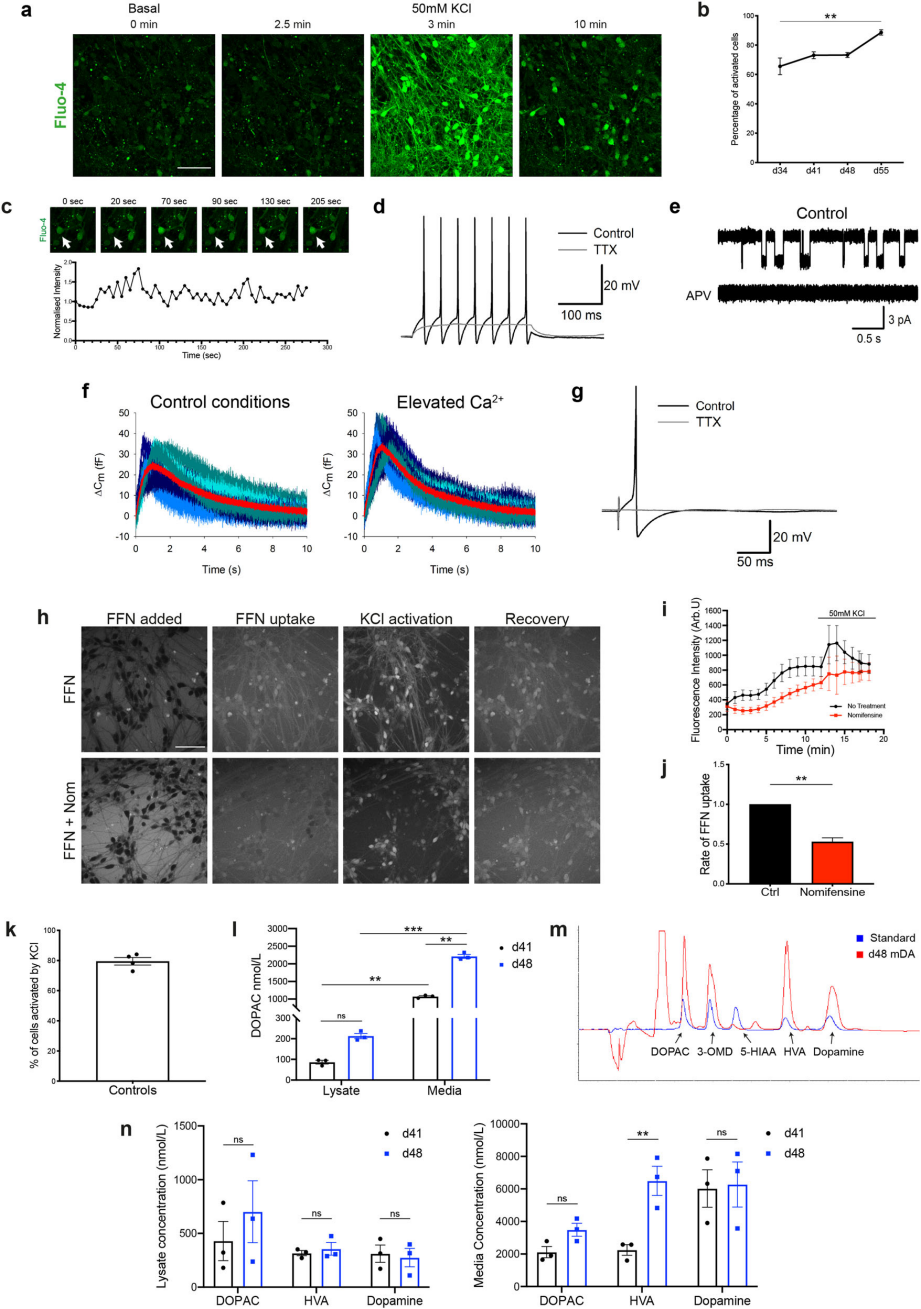
Functional characterisation of mDA neurons. **a** Representative time series images of mDA neurons at day 55 of differentiation, in response to KCl (scale bar = 50 μm). **b** Quantification of the number of cells with calcium response (ns *P* > 0.05, **P = 0.0075, two-way ANOVA). **c** Representative time series images of spontaneous calcium activity at day 41 of differentiation, with the Fluo-4 intensity trace of the highlighted cell (arrow) plotted below. **d** APs triggered by current injection in mDA neurons at day 30 of differentiation. 1 mM tetrodotoxin (TTX) suppresses APs. **e** Single-channel openings of NMDA receptors in an outside-out patch excised from mDA neurons at day 30 of differentiation. Top trace: application of 10 mM glutamate + 10 mM glycine triggers single-channel openings. Bottom trace: 50 mM APV suppresses single-channel opening. **f** Changes in whole-cell membrane capacitance confirm elevated intensity of vesicle release in mDA neurons at day 70 of differentiation. Shadows of blue, high noise. Red trace with low noise: averaged trace. Left: control. Right: elevated Ca^2+^ magnifies the effect on membrane capacitance. **g** AP generation in response to field stimulation in mDA neurons at day 105 of differentiation. **h** Representative images showing sequential uptake and stimulation with 50 mM KCl of the DAT fluorescent substrate FFN102 (FFN) in day 41–48 mDA neurons. Lower panels show FFN uptake in the presence of the DAT inhibitor, nomifensine (scale bar = 50 μm). **i** Representative trace showing increase in intracellular FFN with time, as well as response to KCl stimulation. Nomifensine reduces the rate of FFN uptake. Values plotted as ±s.d. **j** Quantification of the normalised rate of FFN uptake (Welch’s *t* test, **P = 0.0024). **k** Quantification of the number of FFN-positive cells once stimulated by KCl. **l** Quantification of the amount of the metabolite DOPAC in basal day 41 (3w) or day (48) old mDA neurons (ns *P* > 0.05, **P < 0.008, ***P = 0.0002, ordinary two-way ANOVA). **m** Example chromatogram from day 48 mDA neuron culture treated with L-Dopa (red trace) against a standard (blue trace) showing the presence or absence (peaks) of the metabolites DOPAC, 3-O-methyldopa (3-OMD), 5-HIAA, HVA, Dopamine. **n** Quantification of the metabolites DOPAC, HVA, and Dopamine in lysate, or media of l-Dopa treated day 41 and day 48 mDA neurons (ns *P* > 0.05, **P < 0.005, ordinary two-way ANOVA). All values are plotted as ±s.e.m unless stated otherwise. All N numbers for each experiment can be found in Supplementary Table 5.

To test the functional properties of the neurons, we first determined whether they expressed voltage-dependent calcium channels (VDCCs). Opening of calcium channels was induced using a high concentration of KCl (50 mM), which depolarises the plasma membrane, opening VDCCs, resulting in a large influx of Ca^2+^ into the neurons. Bath application of KCl induced a cytosolic Ca^2+^ rise in all cultures with ~65% of cells responding after day 34, increasing to over 85% after day 55 of differentiation (day 34 = 65.5% ± 5.7, day 41 = 73.1% ± 2.3, day 48 = 73.2 ± 1.7, day 55 = 88.6% ± 1.8, *P* = 0.0075) (Fig. 3a, b and Supplementary Fig. 4a). After day 41 of differentiation, we observed spontaneous calcium fluctuations in neurons (Fig. 3c), which are a key hallmark of mDA dopaminergic neurons in the substantia nigra and contribute to the cell pace-making activity^23^.

We investigated the electrophysiological characteristics of the mDA neurons. We confirmed the generation of action potentials (AP) activated by a depolarising voltage step, involving the classical tetrodotoxin (TTX)-sensitive voltage-gated sodium channels (Fig. 3d). We next confirmed the presence of excitatory NMDA receptors, shown by the triggered single-channel openings in outside-patches pulled from cell soma upon application of 10 mM glutamate + 10 mM glycine, and fully inhibited by further application of 50 mM APV, a selective antagonist of NMDA receptors (Fig. 3e). Whole-cell voltage-clamp recording of spontaneous postsynaptic activity revealed postsynaptic currents, which were suppressed by 50 mM of the selective antagonist of GABA_A_ receptor, picrotoxin (Supplementary Fig. 4c). This confirms functional synapses, with synaptic transmission mediated by the main type of inhibitory receptors, GABA_A_ receptors and spontaneously released GABA. Lastly, we tested whether the functional network made by mDA neurons could trigger evoked responses via extracellular electrical stimulation. We recorded the stimulus-evoked APs of a classical shape (Fig. 3g). We observed a clear increase between membrane capacitance (C_m_) value under normal Ca^2+^ concentration and after an elevated Ca^2+^ concentration (Fig. 3f), confirming Ca^2+^-dependent presynaptic vesicle release. Thus, the mDA neurons display complex electrophysiological characteristics of neurons, and are able to form functional networks.

Active dopamine transport can be assessed by measuring the uptake of the fluorogenic dopamine transporter (DAT) substrate FFN102 (FFN)^24^. When incubated with FFN, followed by a wash, the dye can be seen inside mDA neurons (Supplementary Fig. 4b). The uptake of FFN in neurons is further activated upon KCl-induced Ca^2+^ influx in the majority of neurons (Fig. 3h, k) (cells activated by KCl = 79.5% ± 2.5). Inhibition of the DAT using the compound nomifensine resulted in a significantly reduced uptake rate of FFN into neurons (No treatment = 1.00 ± 0.00, nomifensine = 0.53 ± 0.05, *P* = 0.0024) (Fig. 3j) (Supplementary Fig. 4d), as well as a reduced KCl stimulated uptake (Fig. 3i). These data suggest that the mDA neurons exhibit dopamine transport.

HPLC with electrochemical detection was used to investigate dopamine metabolism in the mDA neurons. Under basal conditions, mDA neurons released dopamine metabolite 3,4-Dihydroxyphenylacetic acid (DOPAC), which increased with time in vitro (Fig. 3l) (lysate: d41 = 86.5 nmol/L ± 9, d48 = 212.6 nmol/L ± 13, *P* > 0.05; media: d41 = 1072 nmol/L ± 24, d48 = 2212 nmol/L ± 56, *P* = 0.0041). Therefore, the cells were able to synthesise and metabolise dopamine under basal conditions. Following pre-treatment of the mDA neurons with the dopamine precursor, l-3,4-dihydroxyphenylalanine (l-Dopa), we detected the presence of dopamine, and its metabolites (DOPAC, homovanillic acid [HVA]) in day 41 and 48 terminally differentiated neurons (Fig. 3n). The lack of detection of the main serotonin metabolite 5-hydroxyindoleacetic acid (5-HIAA), usually present in neuronal cells that show mixed dopaminergic/serotonergic characteristics^25^, suggests the mDA neurons are specifically dopaminergic in nature (Fig. 3m). In addition, we found no difference in cell death between treatments, ruling out non-specific release of dopamine from cytotoxicity caused by l-Dopa (Supplementary Fig. 4e). Quantification showed that all metabolites were higher in the media compared to the cell lysate (Fig. 3n), with the metabolites DOPAC and HVA being present in the highest amounts in the media of day 48 mDA neurons (DOPAC = 3493 nmol/L ± 401, HVA = 6494 nmol/L ± 898), and the levels of dopamine being consistent between day 41 and 48 neurons (lysate: day 41 = 312 nmol/L ± 80, day 48 = 274 nmol/L ± 12.8; media: day 41 = 6029 nmol/L ± 1153, day 48 = 6277 nmol/L ± 1387, *P* > 0.05). Together, we show that mDA neurons exhibit dopamine metabolism and secretion by day 41 of differentiation.

### Establishing a synucleinopathy PD model from patient-derived hiPSCs with *SNCA* mutations

**Fig. 4:**
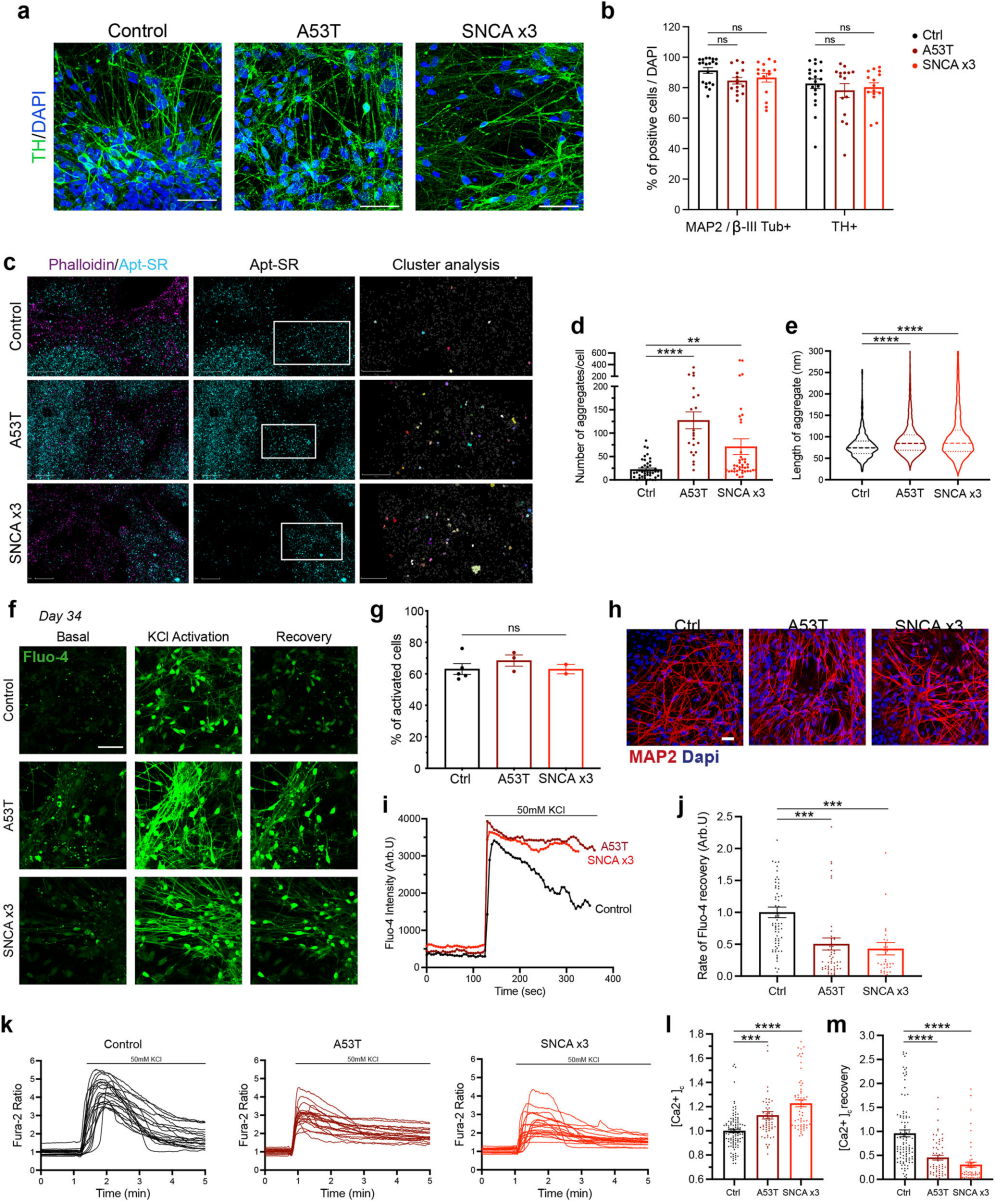
Generation of mDA neurons from hiPSC lines from patients with SNCA mutations display early *α*-synuclein aggregation and calcium dysregulation. **a** Representative ICC images of control, A53T, and *SNCA* x3 mDA neurons showing high TH expression. Scale bar = 50 μm. **b** Quantification of % MAP2 and TH-positive cells after day 41 of differentiation (ns *P* > 0.05, two-way ANOVA). **c** Super-resolved images from control, A53T, *SNCA* x3 day 27 mDA neurons. Left panel shows phalloidin and aptamer. Middle panel shows only super-resolved aptamer binding events. Last panel shows a magnified version of aggregates detected by DBSCAN. Left panel scale bar = 2 μm. Middle panel scale bar = 2 μm. Right panel = 1 μm. **d** Quantification showing the number of aggregates per cell in control, A53T and *SNCA* x3 mDA neurons (ns *P* > 0.05, *P < 0.05, **P < 0.005, one-way ANOVA). **e** Quantification showing the length of all aggregates in control, A53T and *SNCA* x3 mDA neurons represented in a violin plot (****P < 0.0001, one-way ANOVA). **f** Representative time series Ca^2+^ images in response to KCl (scale bar = 50 μm). **g** Quantification of the number of cells with KCl-induced calcium signal at day 34 of differentiation (ns *P* > 0.05, one-way ANOVA). **h** Representative images of neuronal marker expression. **i** Representative single-cell trace in patient mDA neurons. **j** Quantification of the normalised rate of recovery of Fluo-4 after stimulation with KCl (***P = 0.0002, one-way ANOVA). **k** Representative traces showing the Fura-2 ratio in response to 50 mM KCl in day 41 control neurons, A53T neurons, and *SNCA* x3 neurons. **l** Quantification of the basal calcium Fura-2 ratio ([Ca^2+^]_c_) before KCl stimulation (***P < 0.0005, ****P < 0.0001, one-way ANOVA). **m** Quantification of the rate of calcium ([Ca^2+^]_c_) recovery in response to KCl (****P < 0.0001, one-way ANOVA). All values are plotted as ±s.e.m. All *N* numbers for each experiment can be found in Supplementary Table 5.

Mutations in *SNCA* cause an autosomal dominant form of PD (*SNCA*-PD). hiPSCs from patients with a p.A53T mutation, triplication of the *SNCA* locus (*SNCA* x3), their isogenic pairs and healthy donors were differentiated into mDA neurons using the protocol described above. All three mutant lines (two hiPSC lines with the p.A53T mutation, and 1 hiPSC with the *SNCA* x3 mutation) were successfully differentiated into mDA neurons, highlighted by the high enrichment of TH and MAP2 or βIII-Tubulin protein after 41 days of differentiation (Ctrl = 82.6% ± 3.2 TH-positive, 91.3% ± 1.9 MAP2/βIII-positive, A53T = 78.2% ± 4.6 TH-positive, 84.5% ± 2.2 MAP2/βIII-positive, *SNCA* x3 = 80.2% ± 3.0 TH-positive, 86.6% ± 2.9 MAP2/βIII-positive, *P* > 0.05) (Fig. 4a, b). Using RT-qPCR, we first found a 3–4-fold increase in *SNCA* mRNA in the *SNCA* x3 line compared to the control lines at 41 days of differentiation (Supplementary Fig. 5a). The A53T lines exhibited a similar amount of *SNCA* mRNA compared to the control lines (Ctrl = 1.0 ± 0.13, A53T = 1.4 ± 0.04, SNCA x3 = 3.6 ± 0.5, *P* < 0.0001). Using an antibody that recognises total *α*-synuclein, we detected *α*-synuclein protein expression across all lines at day 48 of differentiation, with a significant increase in the expression of *α*-synuclein in the *SNCA* x3 line, in fluorescence intensity and area (Supplementary Fig. 5b–e).

### *α*-synuclein aggregation and abnormal calcium signalling are early and persistent phenotypes in *SNCA* PD mDA neurons

During disease, monomeric α-synuclein aggregates to form small soluble oligomers, with the accumulation of *β*-sheet structure, and later fibrils^26,27^. We investigated whether *SNCA*-PD mDA neurons exhibited early aggregate formation using a single-stranded oligonucleotide aptamer, which binds to *β*-sheet-rich protein structures containing *α*-synuclein^28^. Due to the small size of oligomers (often below the diffraction limit of light ≈250 nm), we employed a previously described super-resolution technique, aptamer-based DNA-PAINT (point accumulation in nanoscale topography) (AD-PAINT) to visualise and quantify *α*-synuclein oligomers. This consists of an aptamer probe that is conjugated to an oligonucleotide sequence which is recognised by the fluorophore-conjugated DNA imaging strand^28^. To demonstrate the specificity of the aptamer for alpha-synuclein aggregates, we performed aptamer-based DNA-PAINT on neuroblastoma cells pre-treated with *α*-synuclein preformed fibrils (PFFs). Seeding in the neuroblastoma cells results in aptamer detection of increased numbers of clusters, which are larger (in length and area) compared to unseeded cells. (Supplementary Fig. 6a, b). After day 27 of differentiation, mDA neurons were incubated with the aptamer, as well as the actin probe, Phalloidin-647. Using a combination of dSTORM (direct stochastic optical reconstruction microscopy) and AD-PAINT, we visualised aggregates at single-cell resolution (Fig. 4c and Supplementary Fig. 6c). Performance of a clustering algorithm analysis, DBSCAN, quantified the clusters based on localisations that were within 60 nm of each other and contained at least 15 localisations (to remove non-specific localisations) (last panel, Fig. 4c). As early as day 27 of differentiation, prior to functional neuronal identity, both A53T and *SNCA* x3 cells displayed a significantly higher number of aggregates per cell (Fig. 4d and Supplementary Fig. 6d) (Ctrl = 22 ± 3.1, A53T = 127 ± 18.2, *SNCA* x3 = 71 ± 16.7, *P* < 0.005). In addition, the average length of the aggregates was higher in A53T, and *SNCA* x3 cells (Fig. 4e and Supplementary Fig. 6e) (Ctrl = 80.0 nm ± 0.99, A53T = 94.9 nm ± 0.83, *SNCA* x3 = 105.2 nm ± 1.32, *P* < 0.0001). In order to rule out non-specific binding of oligonucleotides, cells were incubated with only the imaging strand. The number of single-molecule localisations was low when incubated with only the imaging strand and was significantly increased upon the addition of the aptamer (Supplementary Fig. 6f–h), confirming that the majority of localisations detected are due to the aptamer binding to its target.

Calcium dysregulation is one of the key phenotypes related to α-synuclein aggregation^19,30^. We investigated whether physiological calcium responses were altered by the presence of *SNCA* mutations. We stimulated hiPSC-derived *SNCA* PD mDA neurons with KCl (50 mM) to induce the VDCC-mediated cytosolic calcium signal of neurons at various stages of mDA differentiation. By day 34 differentiation, a similar proportion of control and *SNCA*-PD neurons (60–70%) showed a cytosolic Ca^2+^ increase in response to KCl using the Fluo-4 probe (Ctrl = 63.1% ± 3.5, A53T = 68.4% ± 3.6, *SNCA* x3 = 63.0% ± 3.0, *P* > 0.05) (Fig. 4f–h). Neurons harbouring *SNCA* mutations displayed a significantly impaired recovery of cytosolic Ca^2+^ in response to KCl from day 34 of differentiation (Ctrl = 1.00 ± 0.08, A53T = 0.50 ± 0.10, *SNCA* x3 = 0.43 ± 0.10, *P* < 0.0005) (Fig. 4i, j). At day 41 of differentiation, using the ratiometric calcium indicator, Fura-2, both A53T and *SNCA* x3 mDA neurons displayed higher basal [Ca^2+^]_c_ levels (Ctrl = 1.00 ± 0.01, A53T = 1.13 ± 0.03, *SNCA* x3 = 1.23 ± 0.03, *P* < 0.0005) (Fig. 4k, l). In addition, there was a delayed calcium recovery rate of KCl-induced [Ca^2+^]_c_ in both A53T and *SNCA* x3 mDA neurons (Ctrl = 0.96 ± 0.07, A53T = 0.45 ± 0.05, *SNCA* x3 = 0.31 ± 0.05, *P* < 0.0001) (Fig. 4m), as well as a significantly reduced KCl-induced [Ca^2+^]_c_ amplitude (Supplementary Fig. 6i).

**Fig. 5:**
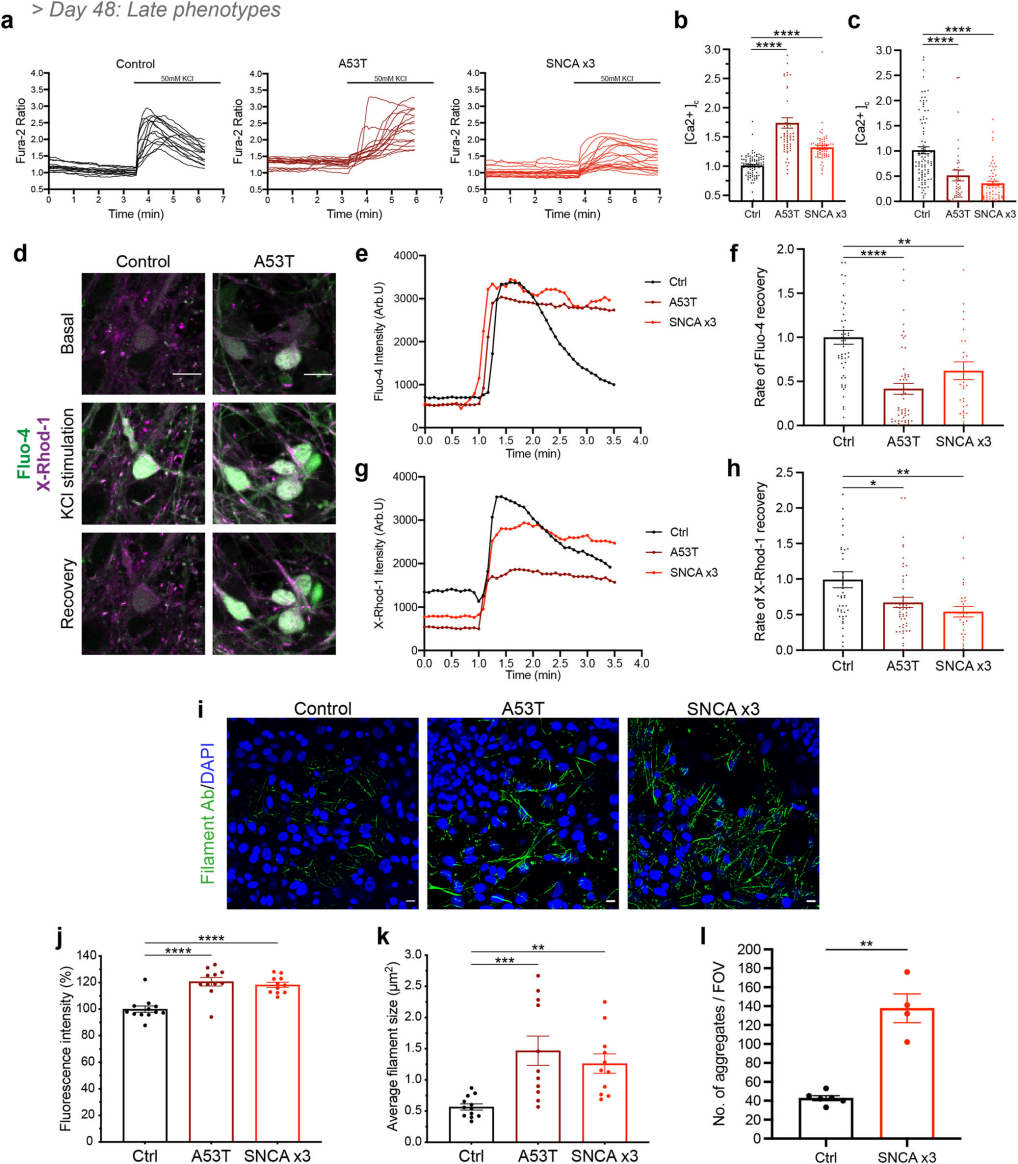
*α*-synuclein aggregation and calcium dysregulation persist in older mDA neurons. **a** Representative traces showing the Fura-2 ratio in response to 50 mM KCl at day 48 of differentiation in control neurons, A53T neurons, and *SNCA* x3 neurons. **b** Quantification of the basal calcium ratio ([Ca^2+^]_c_) before KCl stimulation (****P < 0.0001, one-way ANOVA). **c** Quantification of the rate of calcium ([Ca^2+^]_c_) recovery in response to KCl (****P < 0.0001, one-way ANOVA). **d** Representative time series snapshots of >day 48 control and A53T neurons loaded with Fluo-4 (green) and X-Rhod-1 (magenta) (scale bar = 10 μm). **e** Representative single-cell trace showing delayed recovery of Fluo-4 after KCl stimulation in patient mDA neurons. **f** Quantification of the normalised rate of recovery of Fluo-4 after stimulation with KCl in >day 48-old neurons (**P = 0.003, ****P < 0.0001, one-way ANOVA). **g** Representative single-cell trace showing delayed recovery of X-Rhod-1 after KCl stimulation in patient mDA neurons. **h** Quantification of the normalised rate of recovery of X-Rhod-1 after stimulation with KCl (*P < 0.05, **P < 0.005, one-way ANOVA). **i** Representative ICC images showing the expression of aggregated forms of α-synuclein recognised by a conformation-specific antibody, at day 62 of differentiation. Scale bar = 10 μm. **j** Quantification of the normalised fluorescence intensity of aggregated forms of alpha-synuclein (****P < 0.0001, one-way ANOVA). **k** Quantification of the average puncta size of the aggregated alpha-synuclein (**P = 0.0082, ***P = 0.0007, one-way ANOVA). **l** Quantification showing the number of aggregates per field of view (FOV) from mDA neuronal lysate at day 62 (**P < 0.005, Welch’s *t* test). All values plotted as ±s.e.m. All *N* numbers for each experiment can be found in Supplementary Table 5.

To determine if these phenotypes persist in older mDA neurons (>48 days of differentiation), [Ca^2+^]_c_ was investigated in >day 48 mDA neurons. Both A53T and *SNCA* x3 mDA neurons displayed a higher basal [Ca^2+^]_c_ (Ctrl = 1.01 ± 0.02, A53T = 1.74 ± 0.09, *SNCA* x3 = 1.32 ± 0.03, *P* < 0.0001) (Fig. 5a, b), as well as a delayed recovery following KCl stimulation (Ctrl = 1.02 ± 0.07, A53T = 0.51 ± 0.11, *SNCA* x3 = 0.36 ± 0.04, *P* < 0.0001) compared to control neurons (Fig. 5c). In addition, A53T and *SNCA* x3 neurons had a lower amplitude of KCl-induced [Ca^2+^]_c_ rise compared to control neurons (Supplementary Fig. 7c). During cytosolic calcium influx, mitochondria act as the major buffer for calcium, shaping the cytosolic calcium signal and maintaining calcium homeostasis^29^. Upon KCl-induced calcium influx, we observed a concomitant increase in mitochondrial calcium, measured by the fluorescent dye X-Rhod-1 (Fig. 5d). However, the recovery of the mitochondrial calcium signal was significantly delayed in the *SNCA*-mutant neurons compared to controls, suggesting a potential impairment of mitochondrial calcium efflux (Fig. 5e–h) (Fluo-4: Ctrl = 1.00 ± 0.08, A53T = 0.42 ± 0.06, *SNCA* x3 = 0.62 ± 0.10, *P* < 0.005) (X-Rhod-1: Ctrl = 0.99 ± 0.11, A53T = 0.67 ± 0.07, *SNCA* x3 = 0.54 ± 0.07, *P* < 0.05). Taken together, these results suggest intracellular calcium dysregulation persists as a late phenotype in older mDA neurons and also affects organellar calcium homeostasis.

As we showed that *α*-synuclein aggregation is an early pathological event along with calcium dysregulation, we investigated if it also persisted as a late phenotype in older mDA neurons, using a range of approaches. Using a conformation-specific antibody that recognises all types of aggregated forms of *α*-synuclein^30^ at day 62 of differentiation, we observed the presence of aggregated filamentous *α*-synuclein in both the A53T and the *SNCA* x3 lines which were significantly increased compared to control neurons (Fig. 5i), confirmed by quantification of fluorescence intensity (Fig. 5j) (Ctrl = 100 ± 2.4, A53T = 121 ± 3.2, *SNCA* x3 = 118 ± 1.9, *P* < 0.0001) and the average size of the aggregate puncta (Fig. 5k) (Ctrl = 0.57 μm^2^ ± 0.05, A53T = 1.47 μm^2^ ± 0.24, *SNCA* x3 = 1.26 μm^2^ ± 0.15, *P* = 0.0082; *P* = 0.0007). In addition, serine-129 phosphorylated *α*-synuclein species, a hallmark of Lewy bodies^31^ was detected in neuronal processes of mDA neurons after day 48 (Supplementary Figs. 6j and 7a, b). We also used the aptamer to detect *α*-synuclein aggregates from day 62 neuronal lysate and confirm a significantly higher aggregate load in *SNCA* x3 neurons (Fig. 5l) (Ctrl = 42.7 ± 2.7, *SNCA* x3 = 137.8 ± 15.3, *P* = 0.0073). Finally, we used an *α*-synuclein aggregate sensitive ELISA and similarly found higher levels of aggregates in A53T and *SNCA* x3 neurons in old mDA neurons relative to their respective isogenic control (Supplementary Fig. 7k).

Collectively, our data demonstrate that, using highly sensitive methods, we were able to detect and measure the formation of *β*-sheet-rich, toxic, oligomeric species. Along with aberrant calcium signalling, they both present as the earliest pathological hallmark in *SNCA* neurons. Temporally, aggregation progresses to form phosphorylated and filamentous aggregates that become detectable by traditional immunocytochemical methods.

### Mitochondrial, lysosomal dysfunction and oxidative stress are late *SNCA*-induced phenotypes

**Fig. 6:**
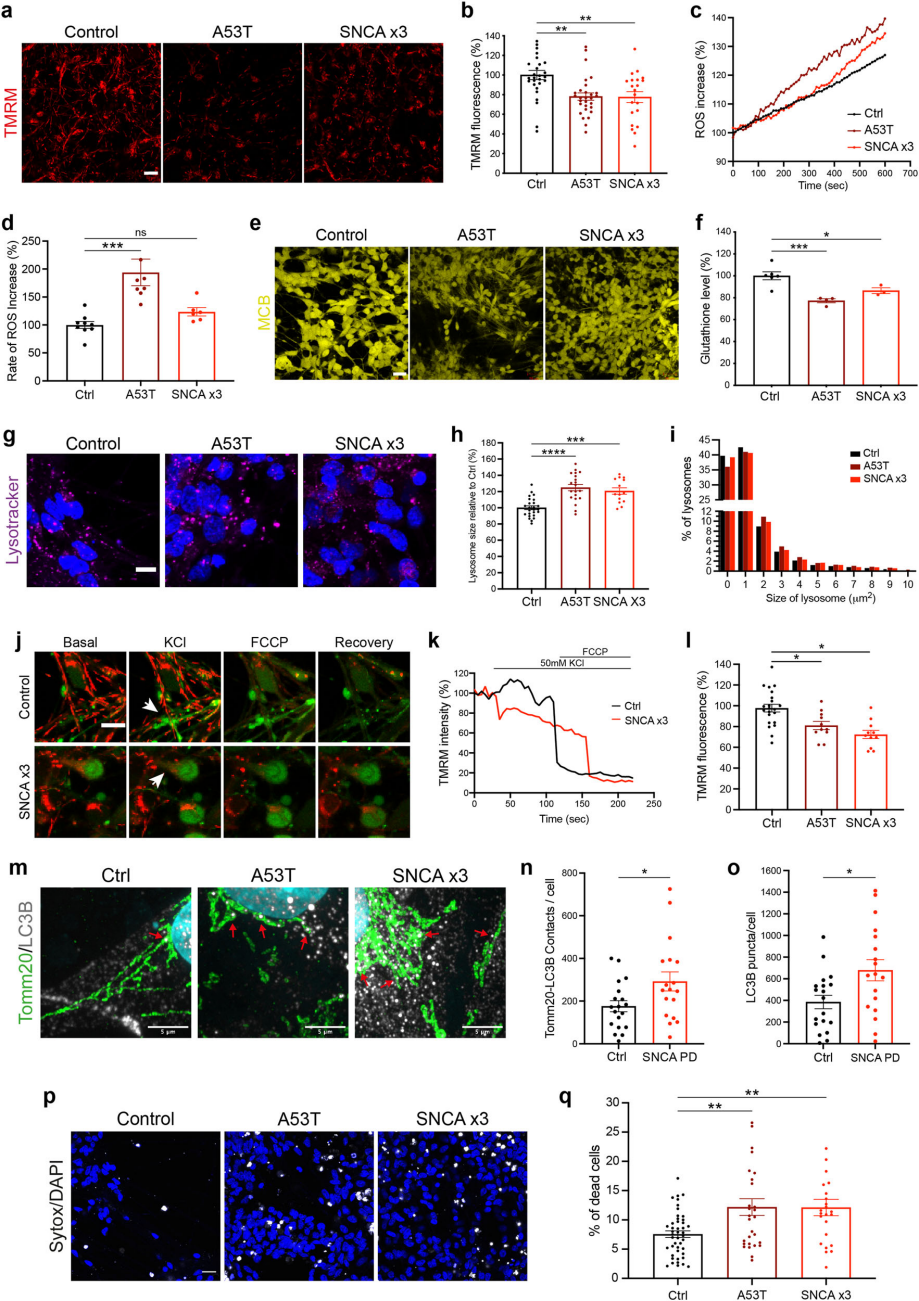
Cellular dysfunction and cell death arise later in *SNCA* PD mDA neurons. **a** Representative live-cell imaging of mitochondrial fluorescence using the lipophilic cationic dye TMRM at day 48 of differentiation. Scale bar = 10 μm. **b** Quantification of the normalised fluorescence intensity of TMRM (**P < 0.005, one-way ANOVA). **c** Trace showing the ratiometric measurement of superoxide generation using dihydroethidium (HEt) at day 48 of differentiation. **d** Quantification of the rate of superoxide generation based on HEt ratiometric fluorescence (ns *P* > 0.05, ***P < 0.0005, one-way ANOVA). **e** Representative live-cell imaging of endogenous glutathione using the fluorescent reporter MCB at day 48 of differentiation. Scale bar = 20 μm. **f** Quantification of the endogenous level of glutathione based on MCB fluorescence (*P < 0.05, ***P = 0.0009, one-way ANOVA). **g** Representative live-cell imaging of lysosomes and nuclear marker Hoechst 33342 at day 48 of differentiation (scale bar = 5 μm). **h** Quantification of the normalised average lysosomal area/size (***P = 0.0002, ****P < 0.0001, one-way ANOVA). **i** Histogram plot showing the percentage of total lysosomes in each set area bin (0–10 μm^2^). **j** Representative time series snapshots of TMRM (red) and Fluo-4 (green) in day 62 old neurons showing the response to KCl and FCCP. The arrow in the control cell highlights polarised mitochondria and calcium response to KCl and FCCP. Arrowhead in the *SNCA* x3 cell highlights KCL-induced TMRM intensity decrease. Scale bar = 10 μm. **k** Representative single-cell trace showing TMRM intensity in response to KCl and FCCP in control and *SNCA* x3 day 62 old neurons. **l** Quantification showing the decrease in TMRM intensity after KCl stimulation in control, A53T and *SNCA* x3 neurons (*P < 0.05, one-way ANOVA). **m** Instant Structured illumination microscopy (iSIM) images of control, A53T, and *SNCA* x3 day 55 neurons probed for mitochondrial marker Tomm20, and the autophagosome marker LC3B. Scale bar = 5 μm). **n** Quantification of the number of Tomm20-LC3B colocalizations per cell (*P < 0.05, Welch’s *t* test). **o** Quantification of the number of LC3B puncta per cell (*P < 0.05, Welch’s *t* test). **p** Live-cell images depicting dead cells in mDA neurons at >day 48 of differentiation using the fluorescent dye SYTOX green (scale bar = 20 μm). **q** Quantification of the percentage of dead cells (**P < 0.005, one-way ANOVA). All values are plotted as ±s.e.m. All *N* numbers for each experiment can be found in Supplementary Table 5.

We assessed the mitochondrial function of *SNCA*-mutant PD neurons using the lipophilic cationic dye TMRM, which accumulates less in depolarised mitochondria upon the loss of mitochondrial membrane potential. Depolarisation of the mitochondria emerges after day 48 of differentiation in *SNCA*-mutant mDA neurons shown by a reduction in mitochondrial membrane potential (Fig. 6a, b) (Ctrl = 100% ± 4.7, A53T = 78.1% ± 3.7, *SNCA* x3 = 77.5% ± 5.5, *P* < 0.005) (Supplementary Fig. 8a). Mitochondrial depolarisation was not evident in early mDA neurons (between days 27 and 48) (Supplementary Fig. 7d, j).

Mitochondrial dysfunction can result in the overproduction of mitochondrial and extra-mitochondrial reactive oxygen species (ROS). We measured the generation of cytosolic ROS using the fluorescent reporter dihydroethidium (HEt), which changes fluorescence emission upon oxidation. After day 48 of differentiation, the rate of superoxide production was significantly higher in the A53T neurons, and approximately 20% higher in the *SNCA* x3 line compared to controls (Ctrl = 100% ± 6.3, A53T = 194.0% ± 23.7, *SNCA* x3 = 123.6% ± 7.5, *P* < 0.0005) (Fig. 6c, d and Supplementary Fig. 8b). At the same neuronal age, we also measured the levels of the endogenous antioxidant glutathione using the Monochlorobimane (MCB) fluorescence indicator, which showed a significant reduction of glutathione in both, A53T and *SNCA* x3 lines compared to the controls (Ctrl = 100% ± 3.7, A53T = 77.2% ± 1.8, *SNCA* x3 = 86.5% ± 2.6, *P* < 0.05; *P* = 0.0009) (Fig. 6e, f and Supplementary Fig. 8c). Oxidative stress was absent in early neurons (Supplementary Fig. 7e–g, j). The combination of increased ROS production together with depleted antioxidant levels suggests that both A53T and *SNCA* x3 mDA neurons exhibit oxidative stress as a late phenotype.

We measured the lysosomal compartment in *SNCA*-PD neurons using LysoTracker Deep red; a cationic fluorescent dye that only accumulates in acidic cellular compartments (lysosomes). We found that both, A53T and *SNCA* x3 neurons after day 48 of differentiation exhibited significantly larger lysosomes compared to the healthy control neurons (Ctrl = 100 ± 2.4, A53T = 125 ± 3.9, *SNCA* x3 = 121 ± 3.9, *P* = 0.0002; *P* < 0.0001) (Fig. 6g, h), which was not detectable in day 27–48 neurons (Supplementary Fig. 7h, j). Control neurons had a higher proportion of smaller lysosomes (mainly between 0–2 μm^2^) compared to the A53T and *SNCA* x3 neurons, which had a higher proportion of larger lysosomes (mostly 2–10 μm^2^) (Fig. 6i and Supplementary Fig. 8e), suggesting that the lysosomes are swollen, implying lysosomal pathology.

### Mitochondrial PTP opening, fragmentation, autophagy and cell death are late phenotypes

To test the consequence of aberrant calcium flux and mitochondrial dysfunction, we loaded *SNCA* and control neurons with cytosolic calcium and membrane potential indicators and tested their response to KCl. In control mDA neurons, KCl induced the opening of the VDCCs, and a cytosolic calcium signal which was associated with the mitochondrial influx of calcium, during which the mitochondria retained their mitochondrial membrane potential (Fig. 6j, k). However, *SNCA* mutant mDA neuronal mitochondria exhibited depolarisation upon calcium influx into the mitochondria, which was induced by the cytosolic calcium signal (Fig. 6k, l) (Ctrl = 97.8 ± 3.7, A53T = 81.0 ± 4.0, *SNCA* x3 = 72.4 ± 3.9, *P* < 0.05), concurrently showing impaired recovery of cytoplasmic calcium (Supplementary Fig. 8f). Rapid depolarisation events in the mitochondria may reflect transient permeability transition pore (PTP) opening, an event triggered by mitochondrial calcium overload that can boost mitochondrial Ca^2+^ efflux. To test the mitochondrial PTP opening threshold in *SNCA* PD, we sequentially applied ferutinin which results in stepwise increases in mitochondrial calcium. PTP opening is measured by a rapid decrease in mitochondrial membrane potential, visualised with TMRM (Supplementary Fig. 8g). In >day 55 *SNCA* x3 neurons, the threshold of mitochondrial PTP opening was significantly reduced compared to control neurons, thereby highlighting mitochondrial damage as a late phenotype (Supplementary Fig. 8h).

Damaged organelles, including mitochondria, as well as protein aggregates, are cleared through the autophagy-lysosomal pathway. Autophagy results in the formation of autophagosomes, which engulf the target, and process them to lysosomes for destruction. We investigated the expression of the autophagosome marker LC3B together with the mitochondrial marker Tomm20 at day 55 of differentiation using a super-resolution approach, structured illumination microscopy (SIM) and iSIM to resolve the contacts between the mitochondria and the autophagosomes. Mitochondria based on Tomm20 immunolabelling in control day 55 neurons maintain an intact network, in contrast to A53T and *SNCA* x3 neurons which exhibit a fragmented mitochondrial network (Fig. 6m and Supplementary Fig. 8i). We also observed a significant increase in the number of colocalisation between Tomm20 and LC3B in all *SNCA*-PD lines compared to control lines (Fig. 6n) (Ctrl = 176 ± 26, *SNCA* PD = 292 ± 45, *P* < 0.05), as well as more LC3B puncta per cell (Fig. 6o and Supplementary Fig. 8j, k) (Ctrl = 385 ± 62, *SNCA* PD = 679 ± 98, *P* < 0.05), suggesting close contact between autophagosomes and the mitochondria and an autophagosome response.

Finally, we examined the viability of the *SNCA* mutant neurons using the fluorescent dye SYTOX^TM^ Green to identify dead cells. We observed a significant increase in cell death in both the A53T and *SNCA* x3 lines compared to the healthy control lines from day 48 onwards (Ctrl = 7.6% ± 0.6, A53T = 12.2% ± 1.4, SNCA x3 = 12.1% ± 1.4, *P* = 0.006; *P* = 0.003) (Fig. 6p, q and Supplementary Fig. 8d). In contrast, at early time points (day 21–41) there was no difference in cell death (Supplementary Fig. 7i, j).

### Late functional consequences of *SNCA* mutations in human mDA neurons

**Fig. 7:**
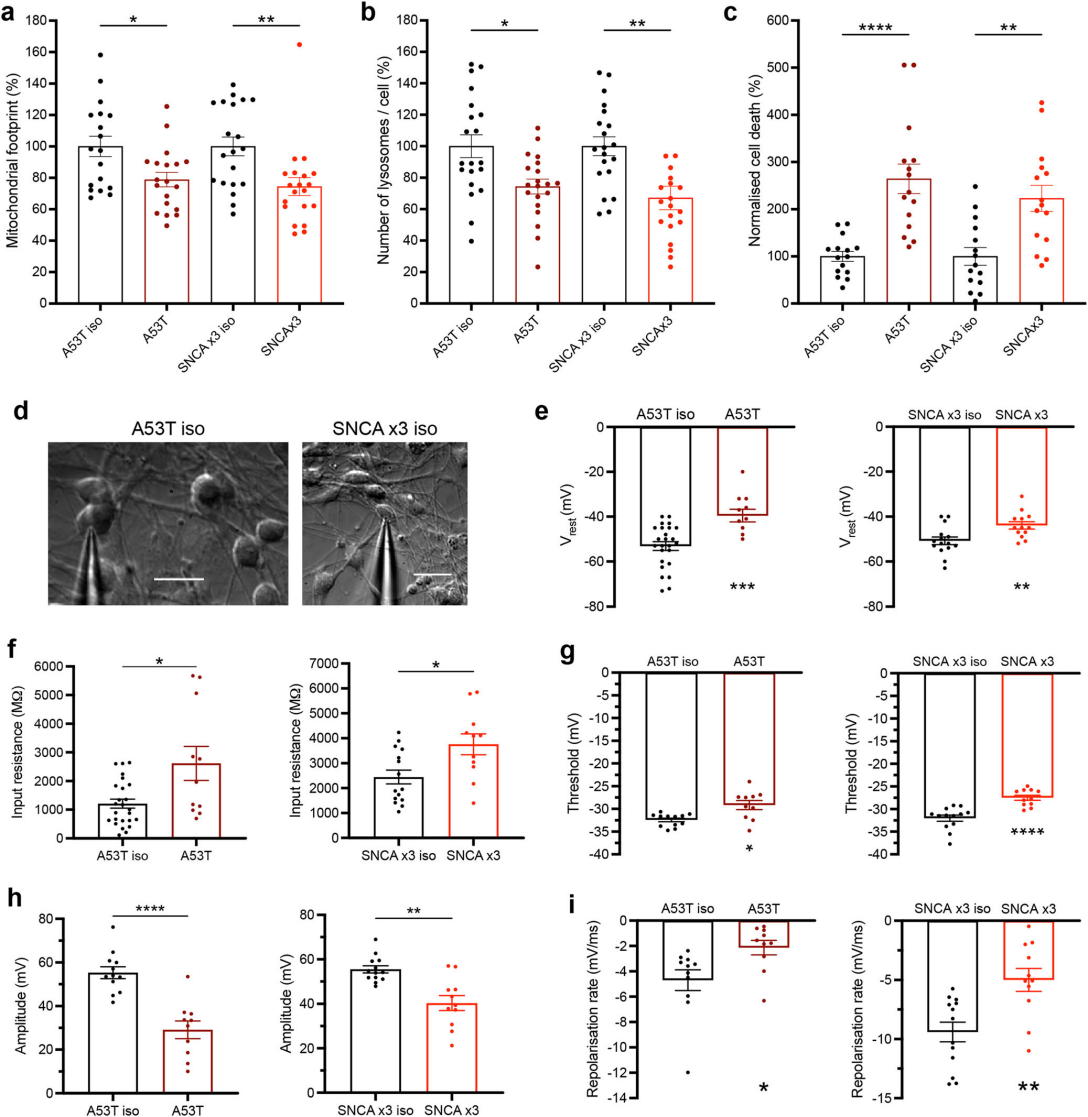
Functional consequences of *SNCA* mutations in human mDA neurons. **a** The normalised basal mitochondrial footprint measurement plotted out in control, A53T, and *SNCA* x3 >day 62 old neurons (*P < 0.05, **P < 0.005, one-way ANOVA). **b** The normalised basal number of lysosomes per cell in controls, A53T and *SNCA* x3 >day 62 old neurons (*P < 0.05, **P < 0.005, one-way ANOVA). **c** The normalised percentage of cell death in control, A53T, and *SNCA* x3 >day 62 old neurons (**P < 0.005, ****P < 0.0001 one-way ANOVA). **d** Representative bright-field images showing >day 70 neurons patched for electrophysiological recordings. Scale bar = 20 μm. **e** Quantification of electrophysiological recordings showing the resting membrane potential in control and A53T neurons, and control and *SNCA* x3 neurons (**P = 0.0068, ***P = 0.0008, Welch’s *t* test). **f** Quantification of electrophysiological recordings showing the input resistance in control and A53T neurons, and control and *SNCA* x3 neurons (*P < 0.05, Welch’s *t* test). **g** Quantification of the threshold for AP generation in control and A53T neurons, and control and *SNCA* x3 neurons (*P < 0.05, ****P < 0.0001, Welch’s *t* test). **h** Quantification of the AP amplitude in control and A53T neurons, and control and *SNCA* x3 neurons (**P < 0.005, ****P < 0.0001, Welch’s *t* test). **i** Quantification of the AP repolarisation rate in control and A53T neurons, and control and *SNCA* x3 neurons (*P < 0.05, **P < 0.005, Welch’s *t* test). All values are plotted as ±s.e.m. All *N* numbers for each experiment can be found in Supplementary Table 5.

To confirm that *SNCA* is the cause of early and late cellular pathology, we generated isogenic controls from A53T and *SNCA* x3 patient lines, and validated our previous findings. After day 55 of differentiation, using TMRM we observed changes in the mitochondrial network in *SNCA*-mutant neurons, with a reduced area/volume occupied by mitochondria (iso-Ctrl of A53T = 100% ± 6.5, A53T = 78.9% ± 4.6, *P* < 0.05; iso-Ctrl of *SNCA* x3 = 100% ± 5.9, *SNCA* x3 = 74.4% ± 5.8, *P* < 0.005.) (Fig. 7a), which was reversed in matching isogenic controls. There was also an increase in the number of lysosomes in both A53T and *SNCA* x3 mDA neurons, which was also reversed in isogenic controls (iso-Ctrl of A53T = 100% ± 7.2, A53T = 74.4% ± 4.7, *P* < 0.05; iso-Ctrl of *SNCA* x3 = 100% ± 6.0, *SNCA* x3 = 67.1% ± 7.5, *P* < 0.005) (Fig. 7b). Similarly, after day 62 of differentiation, there was a significant increase in basal cell death in both *SNCA* PD lines compared to their matching isogenic line (iso-Ctrl of A53T = 100% ± 10.7, A53T = 264.4% ± 31.3, *P* < 0.0001; iso-Ctrl of *SNCA* x3 = 100% ± 18.8, *SNCA* x3 = 222.9% ± 27.6, *P* < 0.005) (Fig. 7c).

We performed patch-clamp recordings in *SNCA*-mutant mDA neurons after day 70 of differentiation (Fig. 7d). In whole-cell configuration, *SNCA* PD mDA neurons displayed a prominently depolarised resting membrane potential (*V*rest) compared with their age-matched isogenic controls (A53T = –39.55 ± 2.84 mV, A53T isogenic = –53.02 ± 1.94 mV, *P* = 0.0004, *SNCA* x3 = −43.92 ± 1.62 mV, *SNCA* x3 isogenic = –50.80 ± 1.69 mV, *P* = 0.0072) (Fig. 7e). However, there was no significant difference in other passive membrane properties of hiPSCs between the mutant and isogenic mDA neurons, such as capacitance (A53T isogenic = *C*_m_: 39.91 pF, A53T = 33.12 pF, *P* = 0.19) and the time constant (A53T isogenic = τ_m_: 99.52 ms, A53T = 100.32 ms, *P* = 0.957) (Supplementary Fig. 9a–c). The similarity of these parameters indicates a similar maturation of biophysical properties between the neuronal groups, with or without *SNCA* mutations. However, the *SNCA*-mutant mDA neurons displayed a significantly increased input resistance, a parameter indicating membrane conductance (A53T *R*_in_: 2.616 ± 0.592 GΩ, A53T isogenic = 1.209 ± 0.158 GΩ, *P* = 0.0415, *SNCA* x3  = 3.758 ± 0.418 GΩ, *SNCA* x3 isogenic = 2.443 ± 0.277 GΩ, *P* = 0.017) (Fig. 7f).

To address how *SNCA* mutations affect neuronal firing capacity, we next carried out recordings from mDA neurons in the current mode. Electrophysiology revealed an impaired firing in both *SNCA* PD neurons. All tested AP parameters were severely distorted in mutant neurons compared with their isogenic control. Firstly, the threshold for an AP spike was depolarised compared with that in an isogenic control (a depolarising shift in A53T = ~3.3 mV, *P* = 0.0108, *SNCA* x3 = ~4.5 mV, *P* < 0.0001) (Fig. 7g), and the AP was significantly reduced in both *SNCA* PD lines (a drop in the amplitude by ~90%, *P*  <0.0001 in A53T neurons and ~37.6%, *P* = 0.0011 in *SNCA* x3 neurons) (Fig. 7h), as well as a decreased AP overshoot (Supplementary Fig. 9g, h). In addition, the spike kinetics were substantially slower (i.e., a drop in the repolarisation rate by 120.5%, *P* = 0.020 in the A53T neurons and 88.1%, *P* = 0.0024 in *SNCA* x3 neurons) (Fig. 7i). Finally, there was also an increased rheobase in *SNCA* mutants – the magnitude of depolarising current required to induce firing. In particular, a two-fold stronger current was needed to bring *SNCA* x3 neurons to firing compared with control (*P* = 0.0026) (Supplementary Fig. 9f). Together, this demonstrates the pathophysiological excitability of PD *SNCA* mDA neurons, with a dramatically changed AP waveform – a profile of changes similar to that observed in other neurodegenerative diseases^32,33^.

**Fig. 8:**
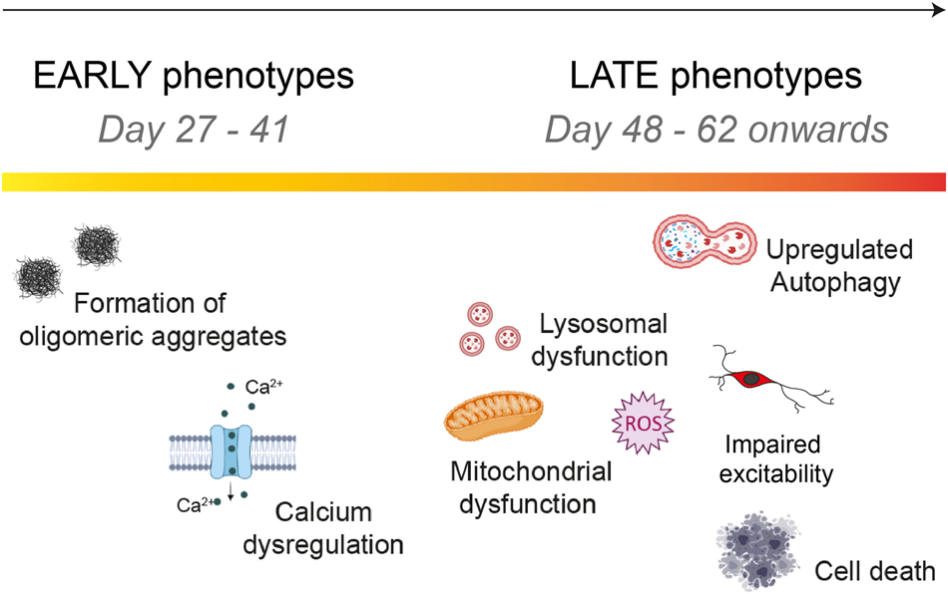
Cellular phenotypes sequentially appear in mDA neurons. A schematic illustration showing temporal sequence of cellular phenotypes in the human PD model. The earliest abnormality is the accumulation of small aggregates with a specific beta-sheet conformation (at day 27 of differentiation). This is followed by another early phenotype which is impaired calcium signalling by day 34 of differentiation. When mDA neurons spend longer in culture, only after day 48, mitochondrial dysfunction, oxidative stress, and lysosomal dysfunction appear as late phenotypes, as well as, upregulated autophagy, impaired excitability and cell death.

In summary, we show that aggregate formation, impaired calcium signalling, mitochondrial and lysosomal homeostasis, and oxidative stress, arise sequentially, alter neuronal excitability and function, and ultimately lead to toxicity to mDA *SNCA*-PD neurons (Fig. 8).

## Discussion

Lineage restriction to diverse cellular fates in the neuraxis is the consequence of interplay of multiple developmental signals, which are regulated in a spatio-temporal manner. In vitro, such lineage restriction can be achieved using a small molecule-based approach to recapitulate ontogeny, drawing insights from mouse developmental biology. In keeping with previous protocols, we initiated neuronal induction via small molecule dual-SMAD inhibition^6^, caudalisation to the midbrain via timed activation of Wnt signalling through a GSK-3*β* inhibitor, and floor-plate ventralization through Shh signalling activated by small molecule agonism. This yielded a population of enriched mDA progenitors, which has been shown to be vitally important in mouse graft outcome^34,35^.

Enriched mDA progenitors were subsequently differentiated into enriched mDA TH-positive neurons (>80%) after day 41, using a combination of two molecules (a ROCK inhibitor and a Notch inhibitor) to allow the mDA NPCs to survive and exit the cell cycle, promoting differentiation into post-mitotic mDA neurons. Diverging from established methods, the mDA NPCs successfully differentiated into enriched mDA neurons without the use of any neurotrophic growth factors^34^, improving the cost-effectiveness of this approach. Single-cell sequencing identified seven neuronal clusters, which expressed key markers of mDA neurons, and a further five clusters with key markers of midbrain NPCs. RNA velocity and latent time analyses^20,36^ revealed the developmental trajectories within the culture, with NPC clusters representing early cell types that were predicted to generate mDA clusters, of varying identities. Furthermore, we identified lists of genes that were associated with these trajectories, which can be further used to study in vitro midbrain neuronal development^37,38^. Our mDA neurons displayed functional properties including calcium channel activity, functional DAT activity, and the synthesis, metabolism and secretion of dopamine. The mDA neurons also display typical neuronal electrophysiological behaviour, pre- and postsynaptic currents, and mature into functional neuronal networks. Differentiating hiPSCs from patients with PD yielded highly enriched mDA neurons of similar molecular and functional identity as control hiPSC-derived mDA neurons, confirming that this differentiation approach may be used to generate models of disease.

In vivo and in vitro models of PD have revealed several putative mechanisms that may cause neuronal toxicity in disease, but virtually all models exhibit several forms of cellular stress occurring simultaneously. We harnessed the developmental nature of the hiPSC system to assess the temporal sequence of events that unfold in the context of *SNCA* mutations, in order to distinguish early, and likely causative events in the cell, from late bystander events. The critical hallmark of synucleinopathy, which is found in the majority of sporadic cases, and therefore the key phenotype for a model to recapitulate, is the detection of aggregated forms of α-synuclein in neurons. Aggregate load in A53T patient lines are reported to be increased due to the mutation^39^, and in *SNCA* x3 patient lines, are higher due to an increase in cellular *α*-synuclein^39^. Aggregates have been reported at day 35 in mDA cultures^40^. We have previously used single-molecule and super-resolution approaches^13^, to detect the formation of the earliest small soluble oligomers outside cells and within cells, and we defined the most toxic aggregate species as a small soluble oligomer with cross *β*-sheet structure^26,30,41^. Here, we applied highly sensitive approaches (super-resolution microscopy) to the *SNCA* mDA neurons to define when aggregation starts. Small oligomeric species of *α*-synuclein were detected early into differentiation (day 27), prior to the molecular and functional specification of midbrain dopaminergic neurons, but after the expression of *SNCA* increased from NPC stage. Both the number and size of these small oligomeric aggregates are higher in *SNCA* A53T and *SNCA* x3 PD lines, and, furthermore, a specific conformation of α-synuclein, that is, the *β*-sheet-rich α-synuclein oligomers, occurs at early stages. These small oligomers are highly hydrophobic and their accumulation is likely to be responsible for the toxicity to cells^27,42,43^. Abnormal aggregation of α-synuclein was a persistent phenomenon, and phosphorylated α-synuclein puncta were apparent using diffraction-limited microscopy from day 41 of differentiation, and the accumulation of phosphorylated forms progressed over time in culture. Finally, conformation-specific antibodies detected fibrillar forms of α-synuclein at later time points, to day 62 of differentiation.

The hydrophobic nature of the oligomer is known to induce a range of cellular stresses, due to its ability to insert and disrupt membranes^44,45^. Notably, the earliest functional phenotype observed in this model is calcium dysregulation at day 34–41 of differentiation. We noted higher basal levels of cytosolic calcium, an increased calcium influx on stimulation, and a delayed calcium recovery in *SNCA*-PD mDA neurons. We have previously shown that the *β*-sheet-rich oligomers and not monomers are able to permeabilise cell membranes due to their lipophilic properties, and induce calcium fluxes, leading to increased cytosolic calcium in response to glutamate and KCl^19,30^. Calcium dysregulation has also been reported in other synucleinopathy models, where it is reported that oligomers interact with receptors and calcium channels^46,47^. Therefore, we suggest that toxic oligomeric species once formed at low concentrations inside the cell due to either an increase in the concentration of *α*-synuclein (*SNCA* x3) or a structural change encouraging aggregation (p.A53T) are responsible for early cellular dysfunction.

Mitochondria are fundamental for cellular function and homeostasis, in particular for buffering cytosolic calcium, and ATP generation, and are a large source of ROS generation^29^. Impairment in calcium fluxes plays an important role in mitochondrial oxidant stress^48^, highlighting the delicate balance between mitochondrial and cellular calcium homeostasis. We previously showed that oligomeric structures of *α*-synuclein interact with ATP synthase, causing mitochondrial dysfunction and early opening of the mitochondrial PTP, which causes neuronal death in *SNCA* x3 neurons^13^. Recent studies using seeding-based models have shown that aggregates interact with and disrupt organelles including mitochondria, autophagosomes and lysosomes, and that the process of aggregate formation drives cellular pathogenesis^49^. Our work complements these findings, and suggests that endogenous unseeded aggregate formation also drives cellular pathogenesis.

Similarly, oligomers have been shown to induce complex I-dependent mitochondrial dysfunction through mitochondrial calcium, which induced swelling and cytochrome C release^50^. A53T hiPSC-derived neurons exhibit an increase in nitrosative, and ER stress^16^, as well as mitochondrial dysfunction^40^. Oligomers further induce the generation of aberrant ROS in both the cytosol and mitochondria^13,18,30^. In this study, we observed abnormalities in mitochondrial function including a reduced membrane potential, abnormal mitochondrial calcium efflux, and fragmentation of the mitochondrial network. Cytosolic calcium fluxes induced rapid mitochondrial membrane depolarisation, reflecting early PTP opening induced by mitochondrial calcium overload. Oxidative stress was evident, based on aberrant generation of superoxide, with a concomitant reduction in glutathione, by day 48 of differentiation.

Autophagy maintains cellular function through lysosome-dependent degradation of damaged organelles or aggregates^51^. In addition to mitochondrial and oxidative stress at a similar time point, we detected a swelling of the lysosomes in the *SNCA* A53T lines. α-synuclein-dependent impairment of lysosomal capacity has been previously reported^17^. In addition to lysosomal alterations, high-resolution microscopy revealed *SNCA*-PD mDA neurons exhibit an increase in autophagosomes that are in close physical contact with fragmented mitochondria, similar to previous studies^52^. Correct electrophysiological properties are vital for neuronal health and function. We found that both *SNCA* mutant lines displayed abnormal AP characteristics, highlighting hypoexcitability as a functional consequence of *SNCA* mutations, similar to other neurodegenerative diseases^32,33^.

Our results support a mechanistic hypothesis that in the disease process, abnormal α-synuclein leads to three major effects in the cell: (i) *β*-sheet-rich oligomeric species disrupt cellular membranes resulting in early cytosolic calcium phenotypes, (ii) oligomers induce disruption of mitochondrial function, oxidative stress and fragmentation of the mitochondrial network, and (iii) a lysosomal response to potentially clear the misfolded α-synuclein. Later, the accumulation of both damaged mitochondria and misfolded protein stimulates an autophagic response in the *SNCA* models, which leads to functional excitability abnormalities.

In summary, we successfully generated highly enriched populations of mDA neurons from hiPSCs, that express mDA markers, functional dopamine transport and form neuronal networks. By enriching the cell of interest in patient-derived lines with *SNCA* mutations, we were able to delineate that the earliest abnormality. Our work leads us to understand more generally how small hydrophobic aggregates with a specific *β*-sheet conformation form early in the neuronal life cycle and can be associated with dysregulation of calcium fluxes and therefore physiological signalling. This is then later associated with cellular stress pathways involving mitochondria and oxidative stress, and affecting protein clearance. Such processes may thus be relevant in all synucleinopathy rather than only the subset of familial PD. Dissecting the temporal sequence of pathological events revealed the first and critical driver of pathogenesis, and subsequent organellar impairment, is the development of toxic protein aggregates.

## Methods

### Human-induced pluripotent stem cell culture

Human-induced pluripotent stem cells (hiPSCs) were maintained in feeder-free monolayers on Geltrex (ThermoFisherScientific) and fed daily with Essential 8 medium (Life Technologies) or mTeSR 1 (StemCell Technologies). When confluent, hiPSCs were passaged using 0.5 mM EDTA (Life Technologies). All cells were maintained at 37 °C and 5% carbon dioxide. The Isogenic control line of *SNCA* A53T was generated using CRISPR/Cas9 editing by Applied StemCell Inc. (USA, project ID: C1729). The isogenic control line of *SNCA* x3 was kindly provided from the Kunath lab and generated using CRISPR/Cas9 editing as published^53^. All hiPSC lines used in this study are described in Supplementary Table 1.

### Midbrain dopaminergic neuron (mDA) differentiation

For all mDA differentiation, hiPSCs were grown to 100% confluency. Media for differentiation was prepared in two separate bottles as: DMEM/F12 + N2 supplement (17502048), MEM non-essential amino acids (5 ml per 1000 ml), β-mercaptoethanol, 5 μg/ml insulin (Sigma) and Neurobasal (phenol red free) + B27 supplement (17504044), l-glutamine (5 ml per 1000 ml), 50 U/ml penicillin–streptomycin, (all from ThermoFisherScientific). Differentiation was triggered by removing old media and replacing it with a 1:1 mix of the DMEM/F12 + N2 and Neurobasal + B27 medias termed “N2B27”. Cells were patterned for 14 days with daily media changes. For the first 2 days, the media was supplemented with the small molecules 5 μM SB431542 (Tocris Bioscience), 2 μM Dorsomorphin (Tocris Bioscience), 1 μM CHIR99021 (Miltenyi Biotec). On day 2, 1 μM Purmorphamine (Merck Millipore) was added. On day 8, CHIR99021, and SB431542 were removed leaving only Dorsomorphin and Purmorphamine in the medium until day 14. Cells were enzymatically dissociated and split on days 4, 10 and 14 using 1 mg/ml of Dispase (ThermoFisherScientific). After patterning, mDA neuronal precursor cells (NPCs) were maintained in N2B27 for 4 days. On day 19, cells were plated onto Geltrex pre-coated Ibidi 8-well chambers (100k/well), clear bottom 96-well plates (50k/well), or 12-well plates (500k/well), and terminally differentiated using N2B27 supplemented with 0.1 μM Compound E (Enzo Life Sciences) and 10 μM Y-27632 dihydrochloride (Rho kinase ROCK inhibitor) (Tocris) from day 20 for the whole duration of terminal differentiation, with two weekly media changes. For super-resolution microscopy, cells were plated on glass-bottom Ibidi eight-well chambers which were pre-coated with poly-d-lysine (PDL) overnight, followed by laminin (Sigma, L2020) in PBS for 1 h. Protocol DOI can be found in Supplementary Table 7.

### Immunocytochemistry (ICC)

For ICC, cells at the desired time point of differentiation had the media removed, followed by one wash in PBS, and fixed with 4% paraformaldehyde for 15 min at room temperature (RT). The paraformaldehyde was then removed, and cells were washed once in PBS. For the LC3B antibodies, samples were incubated in −20 °C methanol after paraformaldehyde fixation. All samples were then blocked for non-specific binding and permeabilized in 5% bovine serum albumin (BSA) (Sigma) + 0.2% Triton X-100 (Sigma) in PBS for 60 min. The primary antibodies were then made up to the desired dilution (Supplementary Table 2) in 5% BSA and applied to the cells overnight at 4 °C. The cells were then washed twice in PBS followed by the application of species-specific, secondary antibodies conjugated to relative AlexaFluor dyes (Supplementary Table 2), at a 1:500 dilution made up in 5% BSA to cells for 60 min at RT in the dark. After secondary antibody incubation, cells were washed once in PBS before being stained with 4′,6‐diamidino‐2‐ phenylindole nuclear stain (DAPI) in PBS for 5 min at a 1:1000 dilution. After DAPI incubation, cells were washed once with PBS before being submerged in a fluorescence mounting medium (Dako), and stored at 4 °C until imaging.

The samples were imaged using Zeiss 880 confocal system with a 40×, 1.4 N.A. oil objective, and a pinhole of one airy units (AU). Between 3 and 5 images were collected per sample, all with a Z projection consisting of five slices, and displayed as a maximum projection. Samples were also imaged using the PerkinElmer Opera Phenix^TM^ High Content Screening System with ×20 and ×40 water objective lenses. A minimum of five fields of view and a Z projection of 3 slices was acquired per well, with the images displayed as a maximum projection. The accompanying software, Columbus^TM^ was used to store and analyse acquired images (https://biii.eu/columbus-image-data-storage-and-analysis-system). The settings for the acquisition of images were kept the same for all samples in the experiment set.

### ELISA assay

To determine the concentration of alpha-synuclein oligomer, cell lysates were mechanically collected at various timepoints and stored at −80 °C. Oligomeric alpha-synuclein was analysed using the Human α-synuclein oligomer (non-A4 component of amyloid precursor) ELISA kit (CSB-E18033h, CUSABIO) according to the manufacturer’s instructions. The levels of oligomeric alpha-synuclein were normalised to the total protein concentration, as determined by the DC Protein Assay (Bio-Rad).

### RNA extraction and quantitative polymerase chain reaction (qPCR)

RNA was harvested from snap-frozen cell pellets using the Maxwell® RSC simply RNA Cells kit (Promega), and the accompanying Maxwell® RSC 48 instrument. After RNA extraction, the RNA concentration and quality using the 260/280 ratio were assessed using the nanodrop. Up to 1 μg of RNA was retro-transcribed into cDNA using the High-Capacity cDNA Reverse Transcription kit (ThermoFisherScientific). The qPCR was performed using TaqMan^TM^ Gene Expression Assay (ThermoFisherScientific). For each gene, TaqMan^TM^ probes were used (Supplementary Table 3) along with the TaqMan^TM^ master mix, and sample cDNA following the manufacturer’s protocol. Samples, along with a minus reverse transcriptase control (-RT) were ran for each gene on the QuantSudio 6 Flex Real-Time PCR System (Applied Biosystems). The -RT served as a negative control, and the gene expression levels were normalised to the housekeeping gene GAPDH following the delta-delta Ct method. Gene expression values were expressed as the normalisation to either hiPSCs or mDA NPCs.

### Single-cell RNA-seq

#### Single-cell generation, cDNA synthesis, library construction and sequencing protocol

After 48 days of differentiation, mDA neurons from 3 control hiPSC lines were washed with PBS once, and then incubated with Accutase (Gibco^TM^) for 5 min to obtain a single-cell suspension. The samples were then diluted 1/3 before usage. The quality and concentration of each single-cell suspension was measured using Trypan blue and the Eve automatic cell counter. Each sample presented a concentration between a 1200–1700 cell/µl and viability ranged between 55 and 68%, samples with a viability above 57% were used for sequencing. Approximately 10,000 cells were loaded for each sample into a separate channel of a Chromium Chip G for use in the 10X Chromium Controller (cat: PN-1000120). The cells were partitioned into nanoliter scale Gel Beads in emulsions (GEMs) and lysed using the 10x Genomics Single Cell 3′ Chip V3.1 GEM, Library and Gel Bead Kit (cat: PN-1000121). cDNA synthesis and library construction were performed as per the manufacturer’s instructions. The RNA was reversed transcribed and amplified using 12 cycles of PCR. Libraries were prepared from 10 µl of the cDNA and 13 cycles of amplification. Each library was prepared using Single Index Kit T Set A (cat: PN-1000213) and sequenced on the HiSeq4000 system (Illumina) using 100 bp paired-end run at a depth of 65–100 million reads. Libraries were generated in independent runs for the different samples.

#### Pre-processing single-cell RNA-seq (scRNA-seq) data

Using the Cell Ranger (RRID:SCR_017344, https://support.10xgenomics.com/single-cell-gene-expression/software/pipelines/latest/what-is-cell-ranger) v3.0.2 Single-Cell Software Suite from 10X Genomics reads were aligned to the human reference genome (Ensembl release 93, GRCh38) (https://support.10xgenomics.com/single-cell-gene-expression/software/pipelines/latest/what-is-cell-ranger). The analysis was carried out using Seurat (RRID:SCR_016341) v3.1.0^54,55^ (https://satijalab.org/seurat/get_started.html) in R-3.6.1 (R Core Team, 2019 (RRID:SCR_001905, http://www.r-project.org/)). The code used for analysis has been deposited online: (10.5281/zenodo.7260558), (https://github.com/strohstern/Transcriptomic_signatures_iPSC_derived_dopamine_neurons). Cells expressing fewer than 200 genes were excluded from the subsequent analysis. Using default parameter within Seurat v3.1.0^54,55^ data for each sample were normalised across cells using the ‘LogNormalize’ function with a scale factor of 10,000. A set of highly variable genes was identified using the ‘FindVariableFeatures()’ function (selection.method = “vst”, nfeatures = 2000). Data were centred and scaled using the ‘ScaleData()’ function with default parameters. Using the highly variable genes, PCA was performed on the scaled data and the first 30 principal components were used to create a Shared Nearest Neighbour (SNN) graph using the ‘FindNeighbors()’ function (k.param = 20). This was used to find clusters of cells showing similar expression using the ‘FindClusters()’ function across a range of clustering resolutions (0.2–1.4 in 0.2 increments). Based on the visualisation of average mitochondrial gene expression across different cluster resolutions using the R package Clustree v0.4.1 (RRID:SCR_016293, https://CRAN.R-project.org/package=clustree)^56^ we selected a clustering resolution of 1.0 to exclude cluster with an average mitochondrial gene expression above 7.5% and, concomitantly, an average number of detected features below 1300.

#### Integration across samples

After filtering of cells/clusters based on mitochondrial gene expression and the number of detected features, we integrated the three samples using the standard workflow from the Seurat v3.1.0 package^55^. After data normalisation and variable feature detection in the individual samples (see above), anchors were identified using the ‘FindIntegrationAnchors()’ function and datasets were integrated with the ‘IntegrateData()’ across 50 dimensions for all detected features in the datasets. We then performed dimension reduction (PC1–45) and cluster identification at resolutions 1.4. After removal of a cluster consisting mostly of suspected doublet cells identified using DoubletFinder^57^, we performed data scaling including cell cycle score regression, dimension reduction (PC1–50) and cluster identification (resolutions 0.4–1.8).

Biomarker of each cluster were identified using Seurat’s ‘FindAllMarkers()’ function using the Wilcoxon rank sum test. We limited the test to positive markers for each cluster in comparison to all remaining cells. The positive marker genes had to be detected in 25% of cells in either of the two groups, with limiting testing further to genes which show, on average, at least 0.25-fold difference (log-scale) between the two groups of cells. Cluster identity was determined using visual inspection focusing on the expression of known marker genes.

#### RNA velocity estimation

For RNA velocity analysis, the spliced and unspliced reads were counted with alevin^58^ as recommended^59^. The count matrices were added to the pre-existing Seurat object which was subsequently used as input into scVelo (v0.2.2, RRID:SCR_018168, https://github.com/theislab/scvelo) to calculate RNA velocity values for each gene of each cell. scVelo was used in the “dynamical” mode with default settings. The resulting RNA velocity vector was embedded into the PCA and UMAP space by translating the RNA velocities into likely cell transitions using cosine correlation to compute the probabilities of one cell transitioning into another cell. We identified driver genes, i.e., those genes that show dynamic behaviour, as those genes with a fit likelihood in the dynamical model >0.3. We also used PAGA^36^ to perform trajectory inference for which directionality was inferred from the RNA velocities.

### Flow cytometry

The protocol for immunolabelling cells for flow cytometry analysis was adapted from a previous study^60^. Cells were washed once with PBS, before being detached into a single-cell suspension using Accutase (Gibco^TM^). A cell suspension of 500 k/ml was prepared in media. Cells were then centrifuged at 200 × *g* for 5 min, and the supernatant was removed. Cell pellet was resuspended gently in 4 ml of 4% paraformaldehyde and briefly vortexed at a low speed before being rotated on a rotation spinner for 10 min at RT. After fixation, samples were centrifuged (200 × *g* for 5 min) and supernatant removed. Cells were resuspended in 2 ml of 0.1% BSA in PBS. After resuspension, cells were filtered through a 70 μm strainer (Miltenyi Biotec) to filter out any cell clumps. Cells were then centrifuged (200 × *g* for 5 min), and the supernatant was removed. Cell pellets were then resuspended in 1 ml of permeabilization/blocking buffer (0.1% Triton X-100, 1% BSA, 10% normal goat serum (Sigma) in PBS), and incubated on a rotation spinner for 30 min at RT. After permeabilization/blocking, cells were centrifuged (200 × *g* for 5 min) and the supernatant was removed. Cells were then resuspended in the primary antibodies (1:200) made up in 0.1% BSA in PBS, and incubated on the rotation spinner for 1 h at RT. After primary antibody incubation, cells were centrifuged (200 × *g* for 5 min), the supernatant removed and washed once in 0.1% BSA in PBS. They were then resuspended in the species-specific secondary antibodies (AlexaFluor 488, 647) at a dilution of 1:500 made up in 0.1% BSA in PBS and incubated in the dark on a rotation spinner for 30 min. After incubation, cells were centrifuged (200 × *g* for 5 min), the supernatant removed and washed once in PBS, followed by incubation with DAPI made up in PBS for 5 min. The DAPI + PBS was then removed, followed by one wash in PBS, before being analysed on the flow cytometer.

The samples were run on the LSRii (BD) cell sorter. Scattering was initially used to discard debris as well as cell doublets and larger clumps. The single-cell population was then gated to include DAPI-positive only cells (negative control). The gating threshold for measured channels was determined using the control lacking the antibody of interest (Fluorescence minus one (FMO) control), for both channels being recorded. Once the parameters had been set, 10,000 cell events were recorded, and data were processed and analysed on FlowJo (RRID:SCR_008520, https://www.flowjo.com/solutions/flowjo).

### Proteomic sample preparation and analysis

In total, 50 µg of lysate from day 55 mDA neurons was taken for proteomic analysis and processed using S-Trap assisted digestion as explained^61^ (protocols.io DOI can be found in Supplementary Table 7). Briefly, proteins were reduced by adding final 10 mM TCEP and incubated the samples on a Thermomixer at 60 °C for 30 min at an agitation of 1200 rpm. Samples were brought to room temperature and alkylated by adding 40 mM Iodoacetamide and incubated on a Thermomixer in dark at room temperature for 30 min at an agitation of 1200 rpm. Further, the SDS concentration was adjusted to 5% vol/vol (final) followed by final 1.2% Vol/vol phosphoric acid was added and processed for the sample clean-up using S-Trap micro columns. Six times the volume of samples, the S-Trap buffer (90% methanol in 100 mm TEABC) was added and loaded on to the S-Trap column for removal of SDS or traces of any reagents by centrifugation at 1000 × *g* at room temperature for a minute. 160 µl of S-Trap wash buffer was added for further clean-up and a total of four washes were given. S-Trap columns were transferred to a new 1.5 ml protein lo-binding Eppendorf tubes for an on-column tryptic digestion, 3.3 µg of Trypsin + Lys-C in 50 µl of 50 mM TEABC buffer was added to the column and subjected to a brief centrifugation (100 × *g* for 30 s) the flow through was transferred back to the column and incubated on a Thermomixer at 47 °C for 1.5 h, followed by an overnight incubation at room temperature. Peptides sequentially were eluted by adding 80 µl of 50 mM TEABC buffer, 0.15% (vol/vol) formic acid and 80% (vol/vol) Acetonitrile in 0.15% formic acid respectively by centrifuging at 1000 × *g* for a minute at each elution step. The eluate was snap-frozen on dry ice and subjected to Speedvac to dryness. Peptides were further stored in −20 freezer until LC-MS/MS analysis. A 2 µg of peptide digest from each sample (*n* = 22) was pooled and subjected High-pH RPLC fractionation as described^61^. To generate a spectral library for Data-Independent Acquisition (DIA) analysis. A total of 46 fractions were prepared for LC-MS/MS analysis.

LC-MS/MS analysis: Peptides were dissolved in LC buffer (3% ACN (vol/vol) in 0.1% formic acid (vol/vol). In total, 2 µg of a peptide from each sample was transferred to LC vial for mass spectrometry analysis on Orbitrap Exploris 480 mass spectrometer in line with Dionex 3000 RSLC nanoliquid chromatography system. Peptides were loaded on to a pre-column (C18, 5 µm, 100 A°, 100 µ, 2-cm nano-viper column # 164564, Thermo Scientific) at 5 µl/min flow rate and separated on a 50 cm analytical column (C18, 5 µm, 50 cm, 100 A° Easy nano spray column # ES903, Thermo Scientific) at 250 nl/min flow rate by applying non-linear gradient of solvent-B (80% ACN (v/v) in 0.1% formic acid (v/v) for about 125 with a total gradient time and run time of 145 min. The data were acquired in data-independent acquisition (DIA) mode. Full MS is acquired at 120,000 resolution *m/z* 200 and measured using Orbitrap mass analyser. The MS2 precursor ions were fragmented using stepped higher energy collisional dissociation (HCD) energies, 25, 28 and 32 and were acquired at 30,000 resolution at m/z 200 and measured using Orbitrap mass analyser. We employed a variable isolation window for MS2-DIA data, a total of 45 vDIA windows were enabled covering the mass range of 350 to 1500 m/z, the details of variable DIA isolation window values are provided in Supplementary table 6. The AGC target for MS1 and MS2 were set at 3000% and ion-injection times were set at 30 ms and 70 ms, respectively.

The spectral library was generated by pooling all samples and generated 46 High-pH RPLC fractions and these were acquired in a Data-dependent Acquisition (DDA) mode on Orbitrap Exploris 240 mass spectrometer. The peptides were separated on a 50 cm analytical column by applying a non-linear gradient for 85 min with a total run time of 100 min. Full MS is acquired at 60,000 resolution at *m/z* 200 and measured using Orbitrap analyzer in the mass range of 375–1500 *m/z*. M/MS data were acquired by employing top speed for 2 s in a data-dependent mode at 15,000 resolution at *m/z* 200 and measured using Orbitrap mass analyser. The quadrupole mass filter isolation window was set to 1.2 *m/z* and precursor ions were fragmented using normalised HCD of 30%. The AGC target for MS was set at 300% and MS/MS was set as standard and maximum ion injection was set in auto mode. The dynamic exclusion duration was set for 45 s.

Mass spectrometry data analysis: The DDA spectral library data was searched with Biognosys Spectronaut pulsar (version: 14.0)^62^ (https://biognosys.com/software/spectronaut/). In total, 46 bRPLC fractions were searched against the Human Uniprot database (Downloaded: 2021/03/16) (Proteome ID: UP000005640). Default search parameters for spectral library generation was used including the data filtered for 1% FDR. The DIA data was searched using Biognosys Spectronaut pulsar software suite (version: 16.0.220606.5300)^62^ (https://biognosys.com/software/spectronaut/) against a spectral library generated from the DDA data and Human Uniprot database (proteome ID: UP000005640). Trypsin as a protease with a maximum of two missed cleavages were allowed and Carbamidomethylation of Cys as a fixed modification and Protein N-ter Acetylation and Oxidation of Met were set as variable modifications. The data was filtered for 1% FDR and the protein group Output files were further processed using Perseus software suite^63^ (RRID:SCR_015753, http://www.perseus-framework.org), and protein copy numbers were estimated using Proteomic ruler plugin within Perseus as described^64^.

### High-performance liquid chromatography (HPLC) and sample preparation

The mDA neurons from three independent hiPSC lines were at day 41 and day 48 of differentiation were incubated in phenol-free “N2B27” medium for 24 h with and without the presence of 80 μM l-Dopa (Sigma D9628). For extracellular metabolites, media after 24 h was mixed 1:1 in 0.8 M ice-cold perchloric acid. Samples were incubated on ice for 10 min, followed by centrifugation at 12,000 × *g* for 5 min at 4 °C. The supernatant was removed and frozen in dry ice for HPLC analysis. For intracellular metabolites, after 24 h, the cells were removed and pelleted by centrifugation at 1200 × *g* for 5 min. The cells were washed once in PBS and lysed on ice using lysis buffer (10 mM Tris (pH 7.4), 1 mM EDTA, 320 mM sucrose in HPLC grade water). The lysate was mixed with 1:1 in 0.8 M ice-cold perchloric acid and incubated on ice for 10 min. The samples were centrifuged at 12,000 × *g* for 10 min and the supernatant was harvested and frozen for HPLC analysis.

Quantification of neurometabolites (DOPAC, 3-OMD, 5-HIAA, HVA and dopamine) was carried out using reverse phase HPLC and an electrochemical detector following a method by^65^. The mobile phase (flow rate 1.5 ml/min) contained 16% methanol, 20 mM sodium acetate trihydrate, 12.5 mM citric acid monohydrate, 3.35 mM 1-octanesulfonic acid, 0.1 mM EDTA disodium and adjusted to pH 3.45 with 12 M hydrochloric acid (HCl). The stationary phase was maintained at 27 °C. The detector electrode was set at 450 mV and screening electrode at 50 mV. 50 µl of the sample was injected and calculated against a 500 nM external standard solution of the 5 compounds of interest made in HPLC grade water acidified with 12 M HCl. Peak areas were quantified with EZChrom Elite™ chromatography data system software, version 3.1.7 (https://www.agilent.com/en-us/support/software-informatics/openlab-software-suite/openlab-cds/ezchromelite320) (JASCO UK Ltd., Great Dunmow, UK).

### Electrophysiology

Visualised patch-clamp recordings from cell cultures were performed using an infrared differential interference contrast imaging system and a Multipatch 700B amplifier controlled by pClamp 10.2 software package (Molecular Devices, USA) (RRID:SCR_011323). For the recordings, a neuronal culture on a glass coverslip was placed in a recording chamber mounted on the stage of an Olympus BX51WI upright microscope (Olympus, Japan). The perfusion solution contained the following (in mM): 119 NaCl, 2.5 KCl, 1.3 Na_2_SO_4_, 2.5 CaCl_2_, 26.2 NaHCO_3_, 1 NaH_2_PO_4_, 2 CaCl_2_, 2 MgCl_2_, 10 glucose (or 22 in some recordings) and was continuously bubbled with 95% O_2_ and 5% CO_2_, pH 7.4. Whole-cell recordings were performed at 32–34 °C; the patch-clamp pipette resistance was 3–7 MΩ depending on particular experimental conditions. Series resistance was monitored throughout experiments using a +5 mV step command, cells with very high series resistance (above 25 MΩ) or unstable holding current were rejected. The intracellular pipette solution for voltage-clamp experiments contained (in mM): 120.5 CsCl, 10 KOH-HEPES, 2 EGTA, 8 NaCl, 5 QX-314 Br^−^ salt, 2 Na-ATP, 0.3 Na-GTP. For current-clamp experiments, the intracellular solution contained (in mM): 126 K-gluconate, 4 NaCl, 5 HEPES, 15 glucose, 1 K_2_SO_4_ × 7 H_2_O, 2 BAPTA, 3 Na-ATP. pH was adjusted to 7.2 and osmolarity adjusted to 295 mOsm. To isolate response of NMDA receptors we added to a perfusion solution: 50 mM picrotoxin, 20 mM NBQX, 1 mM strychnine, 1 mM CGP-55845, 100 mM MCPG, with zero Mg^2+^. To isolate response of GABA_A_ receptors, we added 50 mM APV, 20 mM NBQX, 1 mM strychnine, 1 mM CGP-55845, 100 mM MCPG. All chemicals were purchased from Tocris Bioscience. mDA neurons were tested as a subgroup of the set of generated cultures.

In the whole-cell (immediately after membrane breakthrough), iPSC-derived mDA neurons were recorded for the resting membrane potential (*V*_rest_), membrane capacitance (*C*_m_), the membrane time constant (τ_m_), and input resistance (*R*_in_), measured from the hyperpolarizing squire current pulse steps in current mode, as described earlier^32,33^. To assess the firing capability of the cells, a series of sub- and supra-threshold rectangular current pulses were applied to elicit neuronal firing, with a stepwise-increased stimulus intensity (an increment of 5–10 pA). The *V*_rest_ was set at −60 mV to −70 mV, by injecting a hyperpolarizing bias current where required. The analysis of the AP waveform was performed for the first AP only. The parameters of individual APs were: the spike amplitude (measured from the threshold to the peak), the threshold value, overshoot and the spike width (duration at half-maximal amplitude), the rates of depolarisation and repolarisation phases.

### Live-cell imaging

To measure [Ca^2+^]_c_, cells were loaded with 5 μM of Fura-2 AM in HBSS for 30 min at room temperature, followed by 2x HBSS washes (Invitrogen). Cells were imaged using epifluorescence on an inverted microscope equipped with a 20x fluorite objective. The cells were excited sequentially at 340 and 380 nm using light from a Xenon arc lamp. A time series with 1 or 5 s intervals was performed, establishing basal fluorescence before 50 mM KCl was added to depolarise the membrane. The emitted fluorescence was measured at 515 nm on a cooled camera device (CCD). The fluorescence intensity of the bound and unbound Ca^2+^ was then quantified using ratiometric analysis on Fiji ImageJ (RRID:SCR_002285, http://fiji.sc). [Ca^2+^]_c_ using Fura-2 AM was also imaged on a Nikon Ti2 inverted microscope with Perfect Focus System, an ASI motorised XY stage with piezo Z and an Okolab environmental chamber with a CO^2^ mixer. Images were acquired using an Andor iXon Ultra897 EMCCD camera. Cells were excited with a Cairn FuraLED light engine optimised for 340 and 380 nm with a dichroic mirror T400lp (Chroma) and an emission filter ET510/80 m (Chroma), using a 40 × 1.3 NA S Fluor objective. The microscope was controlled with Micro-Manager v2.0^66^ (RRID:SCR_000415, http://micro-manager.org).

To measure the level of antioxidant, reactive oxygen species (ROS), mitochondrial membrane potential, lysosomal dynamics, calcium uptake, mitochondrial calcium, and cell death, a confocal microscope (ZEISS LSM 710/880 with an integrated META detection system) which has illumination intensity limited to 0.1–0.2% of laser output to prevent phototoxicity was used.

The antioxidant level was measured using a glutathione indicator, 50 μM Monochlorobimane (mBCI, ThermoFisherScientific) which was incubated for 30 min and measured at 420–550 nm excited by a 405 nm laser. To measure mitochondrial membrane potential, cells were incubated with 25 nM tetramethylrhodamine methyl ester (TMRM, ThermoFisherScientific) in HBSS for 40 min and then imaging was acquired using Zeiss LSM 880 confocal microscope. The 560 nm laser line was used to excite and emission was measured above 560 nm. Approximately 3–5 fields of view with Z projections were taken per sample. To measure lysosomal dynamics, cells were incubated with 50 nM LysoTracker^TM^ Deep Red (ThermoFisherScientific), and Hoechst 33342 in HBSS for 40 min and then imaged using Zeiss LSM 880 confocal microscope where the 405 nm, and 647 nm laser line were used to excite Hoechst 33342 and LysoTracker^TM^ Deep Red, respectively. Approximately 4–5 fields of view with Z projections were taken per sample.

Calcium uptake and dynamics were assessed using the dye Fluo-4 AM (ThermoFisherScientific). Cells were incubated with 5 μM Fluo-4 AM in HBSS for 40 min, followed by two HBSS washes. For measuring mitochondrial calcium, cells were incubated with 2 μM X-Rhod-1 AM and Fluo-4 AM in HBSS for 40 min, followed by two HBSS washes. For measuring calcium and mitochondrial membrane potential, cells were loaded with Fluo-4 AM and 25 nM TMRM for 40 min, followed by two HBSS washes. 25 nM of TMRM was then re-added to cells prior to imaging. Live-cell imaging was performed excited by a 488 nm laser and measured at 520 nm. A time series with 5 s intervals was performed to establish basal fluorescence before 50 mM KCl was added to depolarise the membrane and measure fluorescence intensity increase, and recovery.

To measure cell death, live cells were incubated with 500 nM SYTOX^TM^ Green Nucleic Acid Stain (ThermoFisherScientific), and the nuclear marker Hoechst 33342 for 40 min in HBSS. As SYTOX^TM^ Green is impermeable to live cells, only dead cells were stained, whereas Hoechst 33342 labelled all cells. Cells were imaged using the Zeiss LSM 880 confocal microscope where the 405 nm, and the 488 nm laser line were used to excite Hoechst 33342 and SYTOX Green, respectively. Approximately 4–5 fields of view with Z projections were taken per sample.

To measure ROS, (mainly superoxide) a cooled camera device (CCD) was used, and data were obtained on an epifluorescence inverted microscope equipped with a 20× fluorite objective. Cells were loaded with 2 μM dihydroethidium (HEt, Molecular Probe) in HBSS. A time series with 5–10 s intervals was performed. We generated ratios of the oxidised form (ethidium) exited at 530 nm and measured using a 560-nm longpass filter versus the reduced form with excitation at 380 nm measured at 415–470 nm.

To investigate mPTP opening, we used a previously described assay^67^. Briefly, cells were washed 2× with HBSS, and then incubated with 5 μM Fluo-4 AM and 25 nM TMRM in HBSS for 40 min at RT. Cells were then washed 2× in HBSS and 25 nM TMRM in HBSS was to cells again. They were then imaged using the 488 nm laser and 561 nm laser to excite Fluo-4 and TMRM, respectively. A time series was started with 10–20 s intervals over a course of 20 min. To overload cells with calcium to induce mPTP opening, ferutinin was added stepwise after every 2 min, building up the concentration of calcium in the neurons till mPTP opening. The mPTP opening threshold was measured at the point at which rapid loss of TMRM signal occurred, accompanied by a rapid increase in Fluo-4 signal after ferutinin application.

### Fluorescent false neurotransmitter (FFN) live-cell DAT imaging

To measure the presence and activity of the DAT, we utilised the commercially available fluorescent DAT and VMAT2 substrate FFN102 (Abcam, ab120866). To measure the uptake of the FFN102 dye, a field of view was first found using the bright-field settings on a Zeiss LSM 880 confocal microscope. The cells then had 10 μM of the dye in HBSS added and a time series with an exposure every 5 s using the 405 nm laser was started, to measure uptake of the dye into the cells. As a control to confirm specificity, samples in a different well were pre-treated with 5 μM of the DAT inhibitor nomifensine (Sigma) for 10 min in HBSS. Nomifensine was kept in cell solution also after FFN102 was added. Once the dye had entered cells, the cells were depolarised by the addition of 50 mM KCl to observe FFN102 dynamics. Approximately 20 cells were measured per condition/sample and their rate of fluorescence intensity increase was plotted.

### Sample preparation for single-molecule localisation microscopy

For single-molecule localisation microscopy (SMLM), neurons were grown on glass-coated ibidi chambers. Once neurons reached the desired age, they were washed once in PBS, followed by a 15 min fixation in 4% paraformaldehyde + 0.1% glutaraldehyde (both from Electron Microscopy Services) in PBS at RT. The neurons were then reduced in 0.1% sodium borohydride (Sigma) in PBS for 7 min at RT. Cells were then washed 2× in PBS.

To label cells with aptamer and phalloidin, after fixation and PBS washes, cells were permeabilised with 0.25% Triton X-100 in PBS for 10 min at RT. The cells were then blocked in blocking solution (0.1% Triton X-100, 10% normal goat serum (Abcam), 10% salmon sperm DNA (Thermo Fisher Scientific)) in PBS for 2 h at RT. The samples were then incubated with 100 nM of the aptamer (sequence: GCCTGTGGTGTTGGGGCGGGTGCGTTATACATCTA) made up in the blocking solution at 4 °C overnight. After incubation, cells were washed 1x in PBS and incubated with phalloidin-647 (1:400) (Thermo Fisher Scientific) made up in the blocking solution for 1 h at RT. Cells were then washed 1x in PBS and either imaged or incubated with DAPI (1:10000) in PBS for 10 min at RT followed by 2× PBS washes before imaging.

To label aggregates in cell lysate, cells were lysed mechanically in PBS before being centrifuged at 3600 × *g* for 5 min. The supernatant was collected and the protein concentration was quantified using the BCA Protein Assay Kit (Thermo Fisher Scientific). 22 × 40 mm, 1 mm thick glass slides, were cleaned with an argon plasma for 1 h, before 22 × 22 gaskets were affixed to the surface to create a well. The cell lysate was diluted 1 in 10 with filtered PBS (0.02 μm) and 100 nM aptamer (sequence: GCCTGTGGTGTTGGGGCGGGTGCGTTACCACCACCACCACCACCA) and incubated on the surface for 10 min. The sample was then washed off with filtered PBS three times before imaging.

### Single-molecule localisation microscopy

SMLM was performed on a Nanoimager super-resolution microscope (Oxford Nanoimaging Ltd) equipped with an Olympus 1.4 NA 100× oil immersion super apochromatic objective. To ensure efficient blinking for STORM (AF647-tagged phalloidin), the samples were incubated with a blinking induction buffer (B cubed, ONI). Separately to this, AD-PAINT was also employed which relies on the addition of an imaging strand (sequence: CCAGATGTAT-CY3B) to the buffer. 1 nM of the imaging strand was added to the B cubed buffer before imaging. The laser illumination angle was set to 51° for all imaging leading to total internal reflection fluorescence (TIRF). AF647-tagged phalloidin was first imaged for 4000–8000 frames using the 640 nm laser (80% power). After this, 4000–5000 frames at 30% power for the 561 nm laser was used to image and super-resolve the aptamer. Both were recorded at a frame rate of 50 ms. This was done for 2–3 fields of view per line and condition.

For imaging aggregates in neuronal lysate using AD-PAINT, 2 nM of the imaging strand (sequence: GGTGGT-ATTO 655) was added. Images were acquired on Oxford Nanoimager at 20 frames s^−1^, for 8000 frames (20% 635 nm laser power, TIRF).

### Structured illumination microscopy (SIM) and instant SIM (iSIM)

Samples for SIM were cultured on glass-bottom Ibidi 8-well chambers coated with laminin. They were fixed and immunolabelled for intracellular markers as described in the ICC section. SIM was performed on an Elyra PS.1 microscope (Zeiss), using a 40x oil objective (EC Plan-Neofluar 40x/1.30 Oil DIC M27). Images were acquired as 15 × 0.1 µm Z-planes on a pco.edge sCMOS camera, using 5 grid rotations with the 405 nm (23 µm grating period), the 488 nm (28 µm grating period) and the 561 nm (34 µm grating period) lasers. Images were processed and channels aligned using the automatic settings on the ZEN-Black software (Zeiss) (RRID:SCR_018163, http://stmichaelshospitalresearch.ca/wp-content/uploads/2015/09/ZEN-Black-Quick-Guide.pdf).

To perform iSIM, we used an array-scanning confocal super-resolution imaging system (VisiTech International). This consisted of an Olympus IX83 inverted microscope with an ASI motorised XY stage with piezo Z, and images were acquired with a Teledyne-Photometrics BSI Express scientific CMOS camera. All images were acquired using the 150x/1.45NA Apo TIRF objective (Olympus, UAPON150XOTIRF). We used solid-state laser lines: 405, 488, 561, 642 nm with a quad 405/488/561/640 Dichroic mirror (all from Chroma). Images were acquired using the Micro-Manager software and, on-the-fly deconvolution was done using the Mircovolution plugin for Micro-Manager v2.0 (RRID:SCR_000415, http://micro-manager.org).

### Image analysis

ICC images, lysosomal dynamics, cell death and dynamic live-cell imaging were analysed using Fiji ImageJ. For fluorescent intensity readouts, including ICC images, a threshold value was set using control images. This was then used to measure fluorescent intensity, area, and integrated density readouts for all images in the dataset. For each experiment, values were normalised to the average of all the controls in the dataset. To measure puncta including lysosome size, a threshold value was determined using control lines and a mask was generated in order to generate readouts for intensity and area for each puncta in the field of view. These values were then normalised to the average of all control values.

To determine the positive number of TH and/or MAP2/B-III tubulin-positive cells, a nuclear mask was generated using DAPI/Hoechst 33342. This was then overlaid onto the image of interest and the fluorescent intensity readouts for each cell were recorded. A positive cell was defined as being over the fluorescent intensity threshold based on negative cells for each marker.

Ratiometric images were generated using the image calculator. For all dynamic calcium experiments, including Fluo-4, Fura-2, ROS (Het), mPTP, FFN and dynamic TMRM experiments, the Fiji ImageJ plugin “Time Series Analyzer V3” was used. Here, ROIs were randomly selected for each field of view and the plugin was used to generate intensity readouts for each step of the time series. These values for the whole time series were then used to generate traces, and were normalised to the average of control values.

To calculate the mitochondrial footprint as a readout of the mitochondrial network, mDA neurons imaged with TMRM were used and put through the imageJ plugin “Mitochondrial Network Analysis—MiNA” as described^68^, to generate mitochondrial footprint values which were normalised to control neurons.

All graphs and traces were plotted on Prism 8 (GraphPad) (RRID:SCR_002798, http://www.graphpad.com/).

SMLM analysis was performed using the Oxford Nanoimaging Ltd developed online software, CODI (https://pages.oni.bio/codi-advanced-ev-characterisation-made-simple). The super-resolved images are displayed as the output of all super-resolved single-molecule localisations from the entire frame acquisition. Once super-resolution images were uploaded to the software, initially an inbuilt drift correction was performed in order to correct single-molecule localisations in case the sample drifted in the *x* or *y* direction during acquisition. After the drift correction, the number of frames was changed to only include the frames where the relevant fluorophore was imaged. Each localisation was fitted to a 2D Gaussian distribution, and any of those with a standard deviation larger than 250 nm were removed. Finally, any localisations with a precision lower than 20 nm were discarded. Once the filtered super-resolved image was generated, density-based spatial clustering of applications with noise (DBSCAN) was performed on the resulting images. Each cluster in DBSCAN needed to have at least 15 localisations, and each localisation had to be within 60 nm of the other. This was to remove any non-specific aptamer binding, and to only detect aggregates that were quite spatially confined.

AD-PAINT images of cell lysate were analysed using the PeakFit plugin (an imageJ/Fiji plugin of the GDSC Single Molecule Light Microscopy package (http://www.sussex.ac.uk/gdsc/intranet/microscopy/imagej/gdsc_plugins) for imageJ using a ‘signal strength’ threshold of 30 and a precision threshold of 20 nm. The localisations were grouped into clusters using the DBSCAN algorithm in Python 3.8 (RRID:SCR_008394, http://www.python.org/) (sklearn v0.24.2 (RRID:SCR_019053, https://scikit-learn.org/stable/modules/generated/sklearn.decomposition.NMF.html)) using epsilon = 1 pixels and a minimum points threshold of 60 to remove random localisations, which were counted to quantify the number of aggregates per area of coverslip imaged. The code has been deposited on the open-access repository Zenodo 10.5281/zenodo.7123756.

### Statistical analysis

Statistical analysis was performed on Prism 8. To compare two individual groups, a unpaired, two-tailed *t* test was used to generate a *P* value. When comparing more than two individual groups, an ordinary one-way ANOVA was used with a post hoc Tukey test for multiple comparisons between groups. When comparing two individual variables, an ordinary two-way ANOVA was performed, with a correction of the False Discovery Rate or the Tukey’s range test for multiple comparisons between groups. A *P* value below 0.05 was considered to be statistically significant. Results are represented as means ± standard error of the mean (SEM), or standard deviation (SD) where stated in the figure legends. The number of hiPSC lines, number of cells, and the number of neuronal inductions used for each experiment is stated in Supplementary Tables 5 and 6. A ‘*n*’ refers to either: the number of cells for single-cell analysis, or number of fields of view (FOV) for field imaging analyses. A ‘*N*’ refers to either: the number of independent inductions (neuronal induction), or the number of independent hiPSC line (biological repeats). The sizes of the sample for each experiment was selected to ensure that the technical (number of cells, numbers of fields of view, and number of coverslips), and biological (number of hiPSC lines, and number of neuronal inductions) variation was adequately captured, and is listed in Supplementary Table 5.

### Reporting summary

Further information on research design is available in the Nature Research Reporting Summary linked to this article.

## Supplementary information

Supplementary Material

Supplementary table 4

Supplementary table 5

Supplementary table 6

Reporting Summary

## Data Availability

The data that support the findings of this study are available on the open-access repository Zenodo 10.5281/zenodo.7138359. Single-cell RNA-seq raw data were deposited to NCBI Gene Expression Omnibus. The accession code for the data is: GSE213569 (https://www.ncbi.nlm.nih.gov/geo/). Mass spectrometry proteomic raw data and search engine output files were deposited to PRIDE ProteomeExchange^69^ repository, and the data can be accessed using the identifier: PXD035500. The login details are, username: reviewer_pxd035500@ebi.ac.uk. Password: 6YuJLsE7. Protocols used in this study can be found on the repository Protocols.io, and the DOIs can be found in Supplementary Table 7.

## Code availability

The code used for all scRNA-seq and RNA velocity analysis has been deposited online: (10.5281/zenodo.7260558), (https://github.com/strohstern/Transcriptomic_signatures_iPSC_derived_dopamine_neurons). The code used for AD-PAINT lysate cluster analysis has been deposited online: (10.5281/zenodo.7123756).
