# Supplementary material for: Protein aggregation and calcium dysregulation are hallmarks of familial Parkinson’s disease in midbrain dopaminergic neurons
Source: NPJ Parkinsons Dis. 2022 Nov 24;8:162. doi: 10.1038/s41531-022-00423-7 (PMC9691718; doi:10.1038/s41531-022-00423-7)
Supplement: Supplementary file 1 — Supplementary Material [file 41531_2022_423_MOESM1_ESM.pdf]

Supplementary material

Supplementary Figure 1

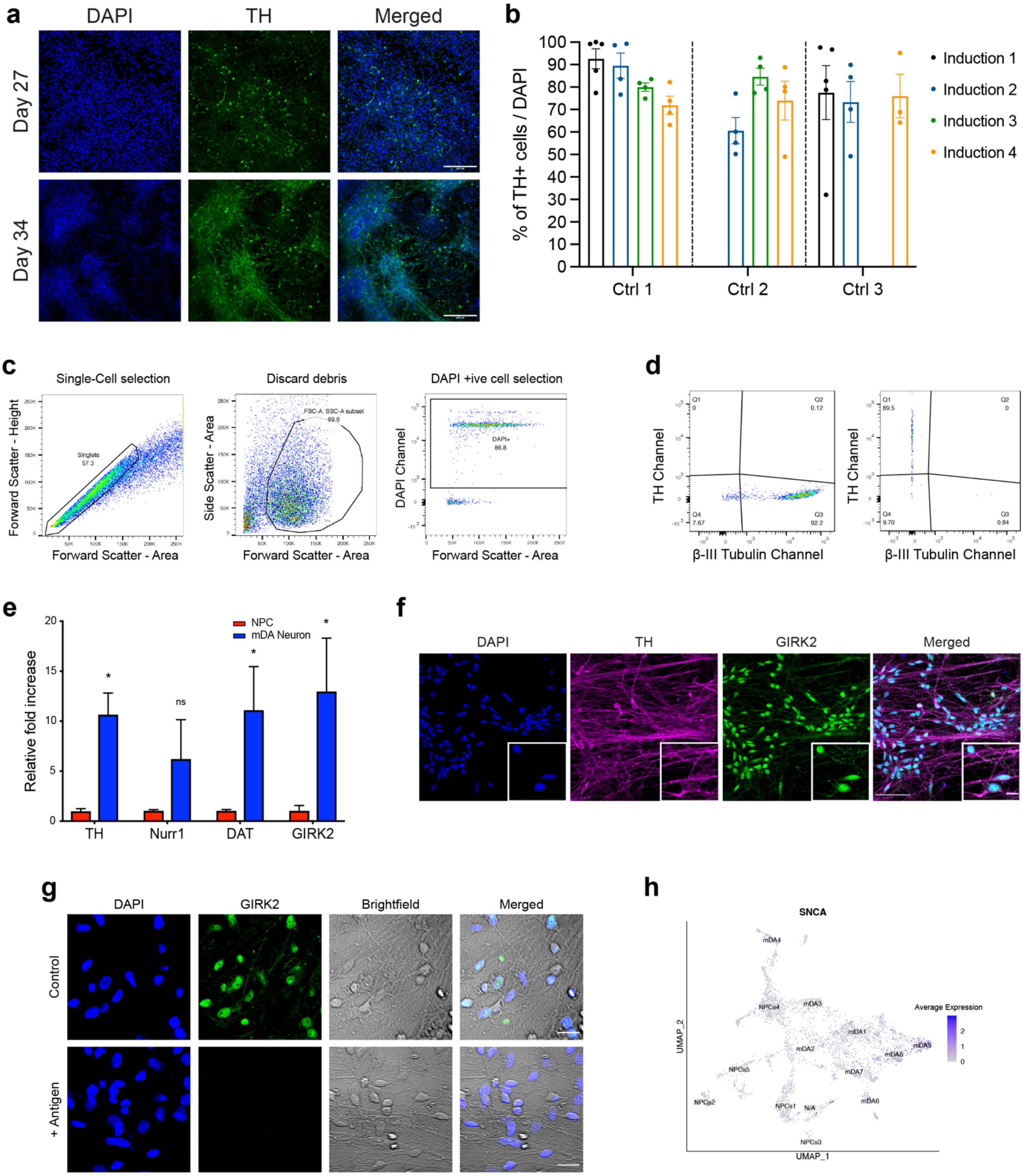

Supplementary Figure 1 – Characterisation of mDA neurons.

(A) Representative ICC images showing the expression of TH increase from day 27 to day 34 of differentiation. Scale bar = 200 µm. **(B)** Quantification of percentage of TH positive cells after 41 days of differentiation across hiPSC lines and inductions showing variability (Ctrl 1: 71.9-92:6%, Ctrl 2: 60.7-84.6%, Ctrl 3: 73.4-77.6%). **(C)** Dot plots showing the selection criteria for flow cytometry analysis. Using the forward scatter, only single cells were selected. Next, in the middle dot plot, cluster was selected discarding any debris. Finally, only the DAPI positive cells were selected. **(D)** Dot plots showing the thresholding gating for the channels measured. Thresholding gates were determined using the fluorescence minus one (FMO) control. In the first dot plot, the TH gate threshold was set by measuring the intensity in the sample with all immunolabelling except TH. The same was done for the β-III Tubulin gate threshold in the right dot plot **(E)** Quantitative PCR showing an up-regulation of mRNA of mature mDA markers *TH, Nurr1*, *DAT*, and *GIRK2* relative to mDA NPCs (*TH* = 10.6 ± 2.2, *Nurr1* = 6.2 ± 3.4, *DAT* = 11.1 ± 4.4, *GIRK2* = 12.9 ± 5.4, ns $p > 0.05$, * $p < 0.05$, two-way ANOVA). **(F)** Representative ICC images showing expression of TH and GIRK2. Scale bar = 50 µm. Smaller image depicts a zoomed-in version of the image showing co-expression of TH and GIRK2. Scale bar = 10 µm. **(G)** GIRK2 Antibody specificity was confirmed using the control antigen. The top panel shows GIRK2 using the antibody as normal. The lower panel shows GIRK2 immunolabelling is abolished when the antibody was incubated with the antigen (GIRK2 sequence). Scale bar = 20 µm. **(H)** A feature plot showing the expression of *SNCA* in all the clusters identified through single-cell RNA-seq at day 48 of differentiation. All values plotted as ±SEM. All N numbers for each experiment can be found in supplementary table 5.

Supplementary Figure 2

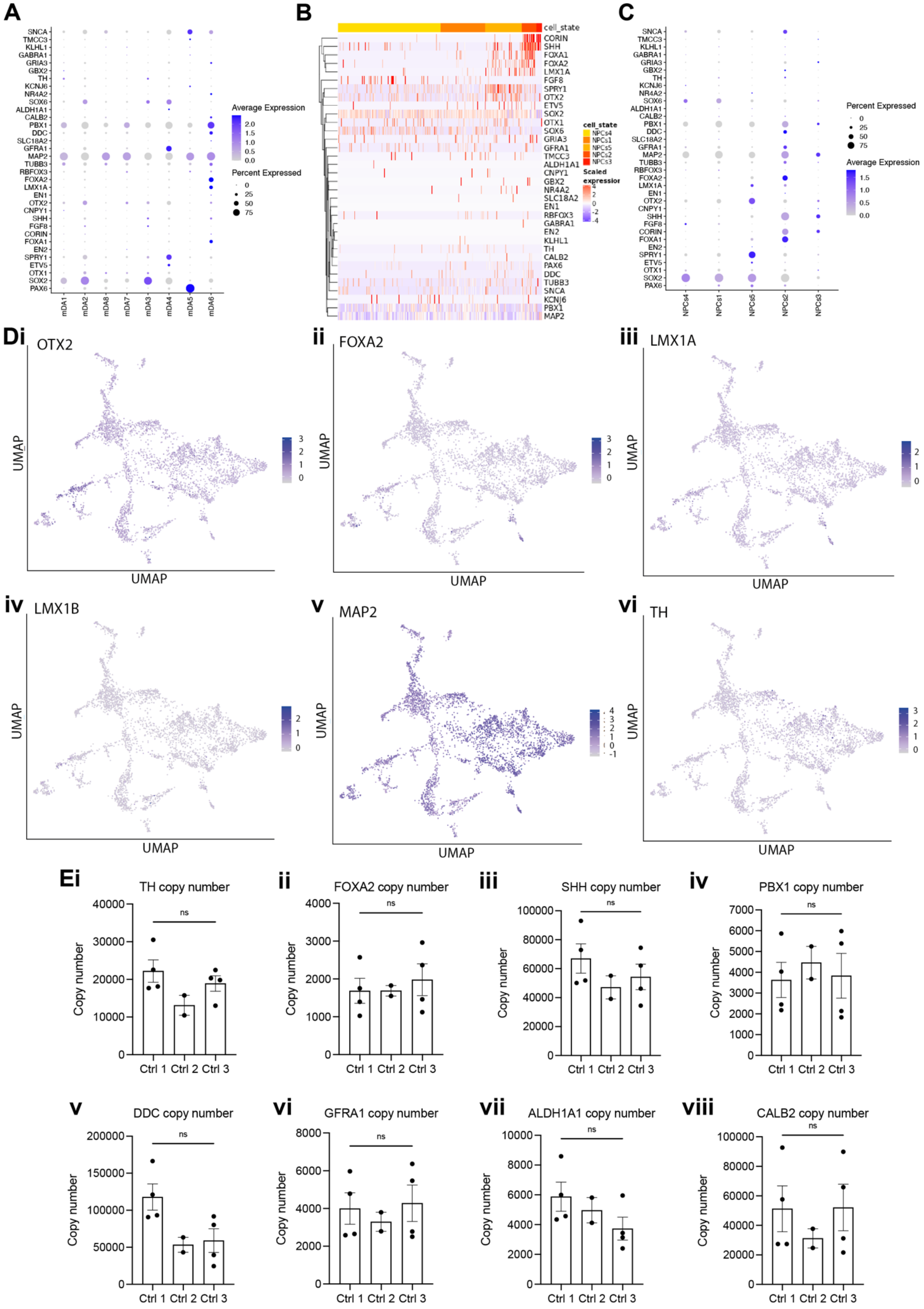

Supplementary Figure 2 – RNAseq UMAP plots for the key markers of mDA neurons.

(A) A dot-plot showing the gene expression profile of the mDA clusters (mDA1-8) identified in single-cell RNA-seq. Genes corresponding to mDA NPC markers and mDA neuron markers are shown. Expression of the genes is coloured based on the expression fold difference and plotted based on the percentage of cells in that cluster expressing the gene. **(B)** A heat map showing the expression of genes in the clusters identified as mDA NPCs (NPCs1-5). Each line represents a cell from that cluster. **(C)** A dot-plot showing the gene expression profile of the mDA NPC clusters. Genes corresponding to mDA NPC markers and mDA neuron markers are shown. **(D)** UMAP feature plots showing percentage gene expression for the key markers **i:** OTX2, **ii:** FOXA2, **iii:** LMX1A, **iv:** LMX1B, **v:** MAP2 and **vi:** TH) of mDA neurons identified from single-cell RNA-seq. **(E)** Bar graphs showing the quantification of copy number proteomic analysis for key mDA proteins: **i:** TH, **ii:** FOXA2, **iii:** SHH, **iv:** PBX1, **v:** DDC, **vi:** GFRA1, **vii:** ALDH1A1, **viii:** CALB2 ($p > 0.05$, one-way ANOVA). All values plotted as ±SEM. All N numbers for each experiment can be found in supplementary table 5.

Supplementary Figure 3

A

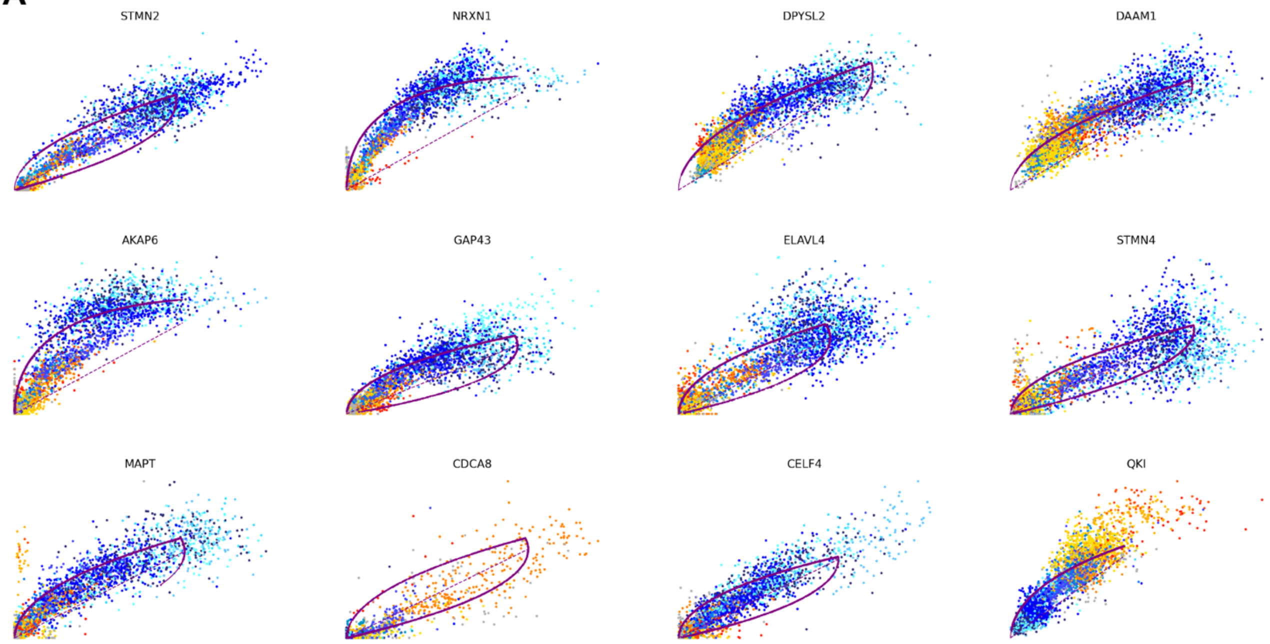

B

	N/A	NPCs1	NPCs2	NPCs3	NPCs4	NPCs5	mDA1	mDA2	mDA3	mDA4	mDA5	mDA6	mDA7	mDA8
**0**	CDCA8	DAAM1	DPYSL2	NDC80	QKI	DAAM1	NRXN1	DAAM1	DAAM1	DAAM1	STMN2	STMN2	STMN2	STMN2
**1**	TM4SF1	DPYSL2	DAAM1	PBK	MEST	MAP1B	AKAP6	NRXN1	DCX	DPYSL2	DPYSL2	AKAP6	NRXN1	NRXN1
**2**	TOP2A	FNBP1L	GAP43	SFRP1	VCAN	MEST	DPYSL2	MAPT	CSRNP3	TM4SF1	DAAM1	GAP43	DPYSL2	GAP43
**3**	CAVIN1	SYT1	ELAVL4	VCAN	HMGA2	CADM2	GAP43	STMN2	FNBP1L	FNBP1L	CSRNP3	DAAM1	AKAP6	DAAM1
**4**	AURKB	QKI	NCAM1	TPX2	MAP1B	PON2	MAPT	DPYSL2	KIF5C	CAV1	GAP43	MEST	CSRNP3	AKAP6

C

	N/A	NPCs1	NPCs2	NPCs3	NPCs4	NPCs5	mDA1	mDA2	mDA3	mDA4	mDA5	mDA6	mDA7	mDA8
**N/A**	0	0.28	0	0.017	0	0	0	0	0	0	0	0	0	0
**NPCs1**	0	0	0	0	0	0	0	0.42	0	0	0	0	0	0
**NPCs2**	0	0	0	0	0	0	0	0.29	0	0	0	0	0	0
**NPCs3**	0	0	0	0	0	0	0	0	0	0	0.18	0.15	0	0
**NPCs4**	0	0	0	0	0	0	0	0.47	0	0	0	0	0	0
**NPCs5**	0	0	0	0	0	0	0	0.33	0	0	0	0	0	0
**mDA1**	0	0	0	0	0	0	0	0	0	0	0	0	0.32	0.35
**mDA2**	0	0	0	0	0	0	0	0	0	0	0	0	0	0
**mDA3**	0	0	0	0	0	0	0.32	0.3	0	0	0	0	0	0
**mDA4**	0	0	0	0	0	0	0	0.39	0	0	0	0	0	0
**mDA5**	0	0	0	0	0	0	0	0	0	0	0	0	0	0
**mDA6**	0	0	0	0	0	0	0	0	0	0	0	0	0	0
**mDA7**	0	0	0	0	0	0	0	0	0	0	0	0	0	0
**mDA8**	0	0	0	0	0	0	0	0	0	0	0	0	0	0

Supplementary Figure 3 – RNA-velocity on mDA neurons.

(A) Phase portraits of some of the driver genes identified in each cluster. Blue dots represent mDA neuron clusters, yellow dots represent NPC clusters. Driver genes for neuronal clusters have higher amounts of neuronal cells at the top of the plot (*STMN2*) whereas driver genes for NPC clusters (*QKI*) have a higher amount of NPC cells at the top. Lines represent the "steady-state" ratio, i.e. the ratio of unspliced to spliced mRNA abundance which is in a constant transcriptional state. *QKI* for example is expressed (steady-state expression) more in NPCs, while it is not expressed in mDA neurons. *ELAVL4* on the other hand is expressed at a steady-state in mDA neurons and is up-regulated in NPCs. **(B)** A table showing the top 5 driver genes for each cluster identified through RNA-velocity. **(C)** PAGA analysis visualised in Figure 2C in a table showing the numeric expression of the transition confidences between clusters. All N numbers for each experiment can be found in supplementary table 5.

Supplementary Figure 4

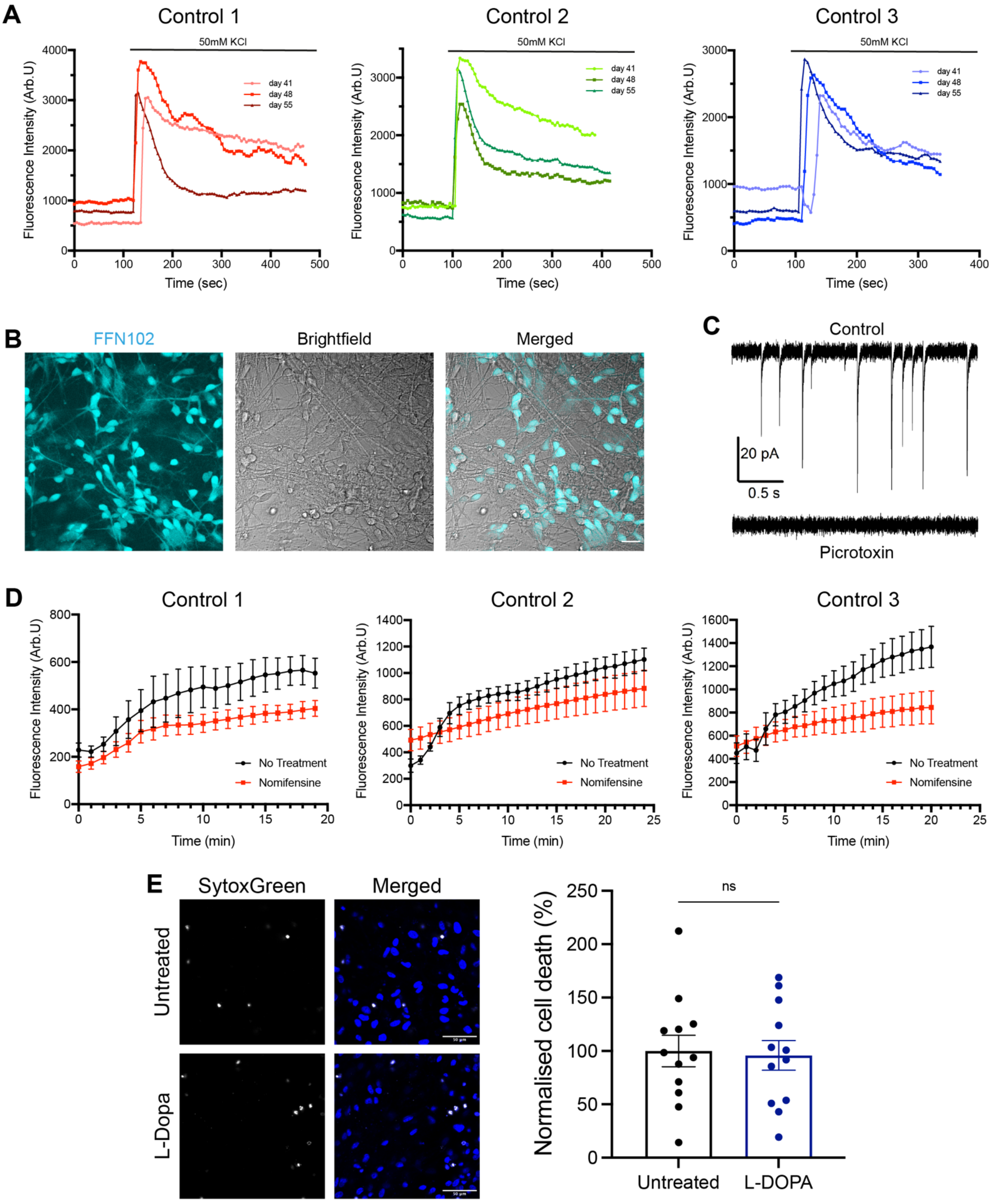

Supplementary Figure 4 – Ca^{2+} and DAT functionality in mDA neurons.

(**A**) Traces showing the Fluo-4 mean fluorescence intensity for each hiPSC control line before and after the addition of 50mM KCl from day 41 to 55 of differentiation. Values plotted as the mean of all cells per time point of differentiation**.** **(B)** Live-cell imaging picture of cells after a 30-minute incubation with FFN, showing the dye enters most mDA neurons. Scale bar = 20 μm. **(C)** Spontaneous postsynaptic activity in mDA neurons at day 70 of differentiation. Top trace: spontaneous postsynaptic currents. Bottom trace: 50 mM of picrotoxin suppresses spontaneous synaptic activity. **(D)** Traces showing the fluorescence intensity of FFN inside cells to measure the uptake of the dye in each hiPSC line tested, in the absence, or presence of DAT inhibitor nomifensine (n = 15-20 cells per condition). Values plotted as ±SD. **(E)** Cell death assay of day 48 old mDA neurons in untreated and 24hr, 80 μM L-DOPA treated samples. Dead cells are indicated with SytoxGreen signal. Bar graph on the right shows the normalised percentage of dead cells in both conditions (Untreated = 100 ± 14.8%, L-Dopa = 95 ± 14.0%, $p > 0.05$ unpaired t test). Scale bar = 50 μm. Values plotted as ±SEM. All N numbers for each experiment can be found in supplementary table 5.

Supplementary Figure 5

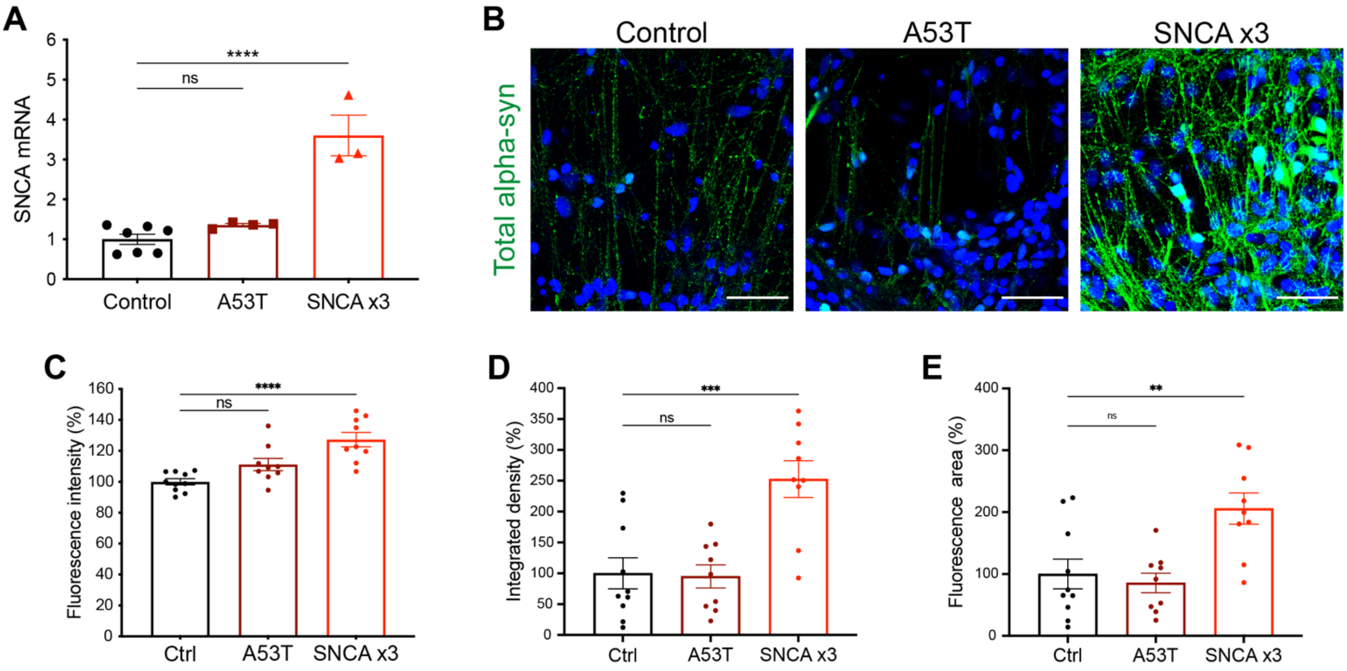

Supplementary Figure 5 – α-synuclein expression in *SNCA* PD mDA neurons.

(A) Quantitative PCR showing relative mRNA expression of *SNCA* in mDA neurons at day 41 of differentiation (ns $p > 0.05$, **** $p < 0.0001$, one-way ANOVA). **(B)** Representative ICC images showing the expression of α-synuclein at day 48 of differentiation. Scale bar = 50 μm. **(C)** Quantification of the relative normalised fluorescence intensity of α-synuclein in all lines (Ctrl = 100 ± 2, A53T = 111 ± 4, *SNCA* x3 = 127 ± 4.7, ns $p > 0.05$, **** $p < 0.0001$, one-way ANOVA). **(D)** Quantification showing the product of the area and fluorescence intensity of α-synuclein (integrated density) in all lines (Ctrl = 100 ±25, A53T = 95 ± 19, *SNCA* x3 = 253 ± 30, ns $p > 0.05$, *** $p = 0.0006$, one-way ANOVA). **(E)** Quantification showing the fluorescent area of α-synuclein in all lines (Ctrl = 100 ±24, A53T = 86 ± 16, *SNCA* x3 = 206 ± 25, ns $p > 0.05$, ** $p < 0.005$, one-way ANOVA). All values plotted as ±SEM. All N numbers for each experiment can be found in supplementary table 5.

Supplementary Figure 6

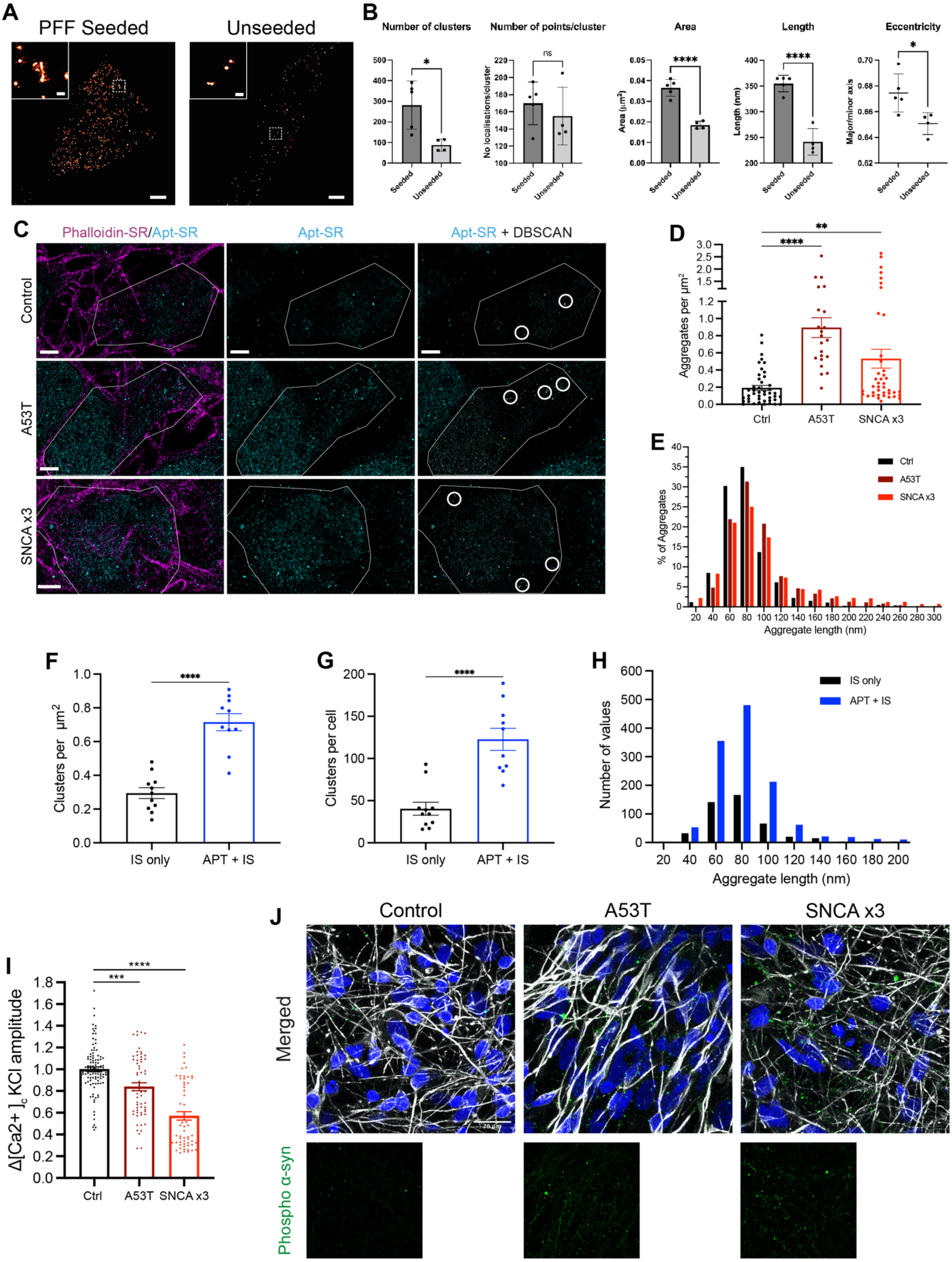

Supplementary Figure 6 – Oligomer and aggregate detection in mDA neurons.

(A) Representative images of SH-SY5Y cells seeded (left) or unseeded (right) with α-synuclein PFFs and imaged using SMLM AD-PAINT showing detectable aggregates. Scale bars: 5 μm (big panel); 500 nm (small panel). **(B)** Quantification of clusters in αSyn PFF seeded vs unseeded SH-SY5Y cells showing number of clusters per cell, localisations per cluster, area, length, and eccentricity of clusters. Each data point represents the mean value per image (ns $p > 0.05$, * $p < 0.05$, **** $p < 0.0001$, unpaired t test). Values plotted as ±SD. **(C)** SMLM images showing phalloidin in magenta and the aptamer in cyan in day 27 mDA neurons. White line shows the ROI of the cell identified. Middle panel shows only the SMLM aptamer. Final panel shows oligomers identified by DBSCAN (in different colours) with example clusters highlighted in circles. Scale bar = 2 μm. **(D)** Graph showing the number of aggregates normalised to the area in each cell expressed as aggregate per μm^2 (Ctrl = 0.19 ±0.03, A53T = 0.89 ±0.12, *SNCA* x3 = 0.53 ±0.11, ** $p = 0.0058$, **** $p < 0.0001$, one-way ANOVA). **(E)** Each aggregate plotted as a percentage of the total population (y-axis) showing the length of each aggregate binned in 20 nm intervals. **(F)** Graph showing the aggregates per μm^2 of each cell when samples were imaged with only the imaging strand (IS) or with prior incubation with the aptamer (IS only = 0.29 ±0.03, APT+IS = 0.72 ±0.05, **** $p < 0.0001$ Welch's t-test). **(G)** Graphs showing the clusters per cell when imaged with only the IS or with prior incubation with the aptamer (IS only = 40.5 ±7.8, APT+IS = 123 ±13.0, **** $p < 0.0001$ Welch's t-test). **(H)** Histogram showing the length of each cluster binned in 20 nm intervals in IS only and the with incubation of the aptamer (n = 400-1300 aggregates per condition). **(I)** Quantification of the change in cytosolic Ca^{2+} amplitude ($\Delta[Ca^{2+}]_c$) after KCl addition in day 41 neurons (Ctrl = 1.00 ±0.02, A53T = 0.84 ±0.04, *SNCA* x3 = 0.57 ±0.04, *** $p = 0.0005$, **** $p < 0.0001$, one-way ANOVA). **(J)** Representative ICC images showing day 48 mDA neurons immunolabelled with neuronal marker MAP2 (grey) and Ser129 phosphorylated α-synuclein. Lower panel shows only phosphorylated α-synuclein signal. Scale bar = 20 μm. All values plotted as ±SEM unless stated otherwise. All N numbers for each experiment can be found in supplementary table 5.

Supplementary Figure 7

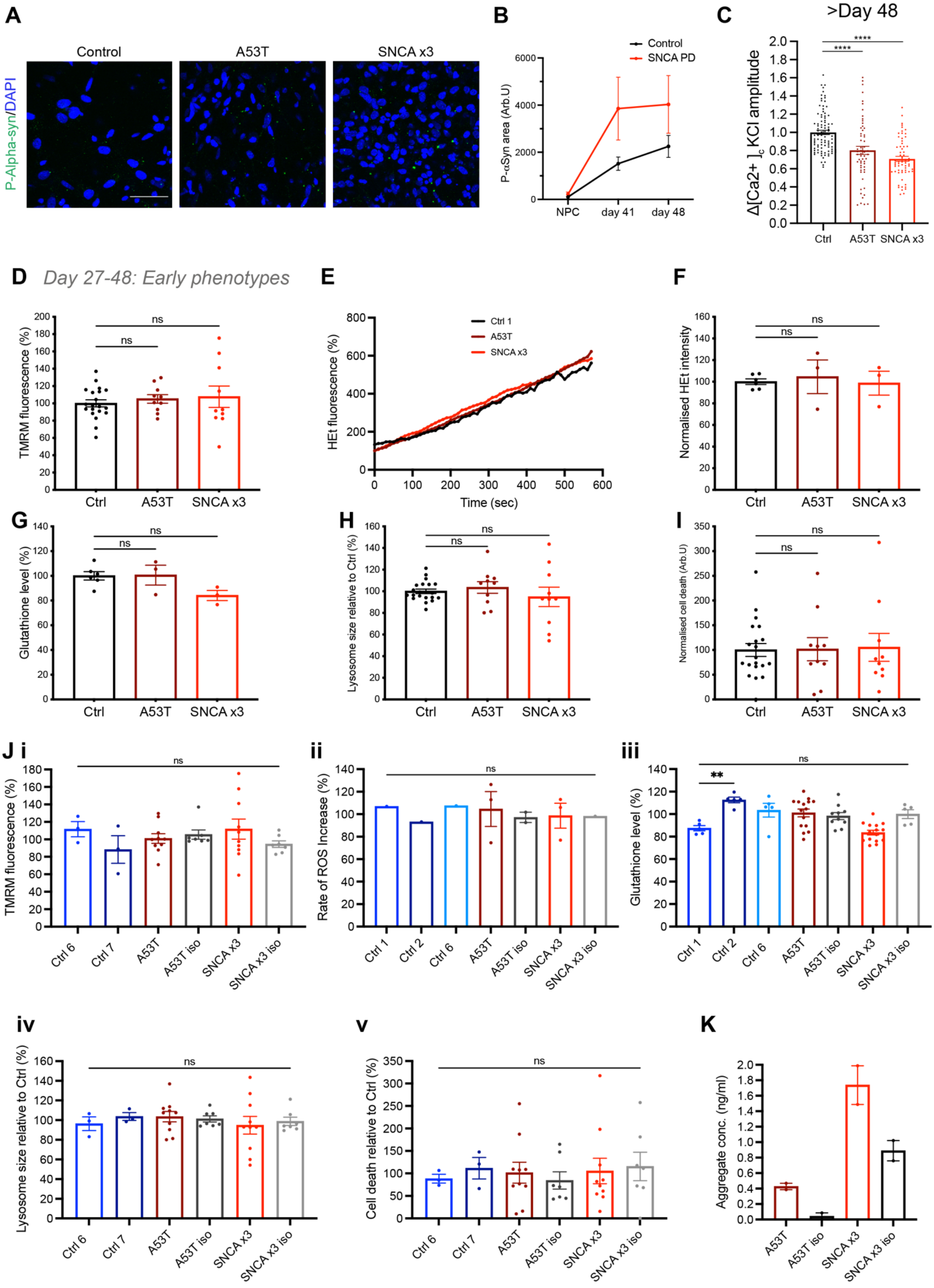

Supplementary Figure 7 – Aggregation, calcium dysregulation and early cellular pathology in mDA neurons.

(A) Representative ICC images showing phosphorylated α-synuclein in green at day 48 of differentiation in control, A53T, and *SNCA* x3 lines. Scale bar = 50 μm. **(B)** Line graph showing the normalised phosphorylated α-synuclein area over time from NPCs through to day 48 of differentiation in control and *SNCA* PD lines. **(C)** Quantification of the change in cytosolic Ca^{2+} amplitude ($\Delta[Ca^{2+}]_c$) after KCl addition in day 48 neurons (Ctrl = 1.00 ±0.02, A53T = 0.80 ±0.05, *SNCA* x3 = 0.71 ±0.03, **** p <0.0001, one-way ANOVA). **(D)** Quantification showing the TMRM fluorescence in early day 27-48 mDA neurons (Ctrl = 100 ± 4.1, A53T = 105 ±5.0, *SNCA* x3 = 108 ±12.4, ns p > 0.05, one-way ANOVA). **(E)** Representative control, A53T and *SNCA* x3 traces showing the HEt fluorescence over time in day 27-48 mDA neurons. **(F)** Quantification showing the rate of ROS increase from the HEt fluorescence in day 27-48 mDA neurons (Ctrl = 100 ±2.7, A53T = 105 ±15.5, *SNCA* x3 = 99 ±11.1, ns p > 0.05, one-way ANOVA). **(G)** Quantification of the Glutathione fluorescence in day 27-48 mDA neurons (Ctrl = 100 ±3.4, A53T = 101 ±8.0, *SNCA* x3 = 84 ± 4.1, ns p > 0.05, one-way ANOVA). **(H)** Quantification showing the normalised lysosome size in day 27-48 mDA neurons (Ctrl = 100 ± 2.1, A53T = 104 ± 5.4, *SNCA* x3 = 95 ±9.0, ns p > 0.05, one-way ANOVA). **(I)** Quantification showing the normalised basal cell death in day 27-48 mDA neurons (Ctrl = 100 ±13.1, A53T = 102 ± 23.4, *SNCA* x3 = 105 ±28.2, ns p > 0.05, one-way ANOVA). **(J)** Graphs showing the each of the early phenotypic assays performed above (day 27-48) split to show each cell line used for **i:** TMRM fluorescence, **ii:** rate of ROS increase (HEt fluorescence), **iii:** Glutathione, **iv:** lysosome size, **v:** basal cell death (** p < 0.005, ns p > 0.05, one-way ANOVA). **(K)** ELISA quantification showing the concentration of aggregated α-synuclein in day 62 old neurons (A53T = 0.43 ± 0.04, A53T iso = 0.04 ± 0.04, *SNCA* x3 = 1.74 ±0.25, *SNCA* x3 iso = 0.89 ±0.13). All values plotted as ±SEM. All N numbers for each experiment can be found in supplementary table 5.

Supplementary Figure 8

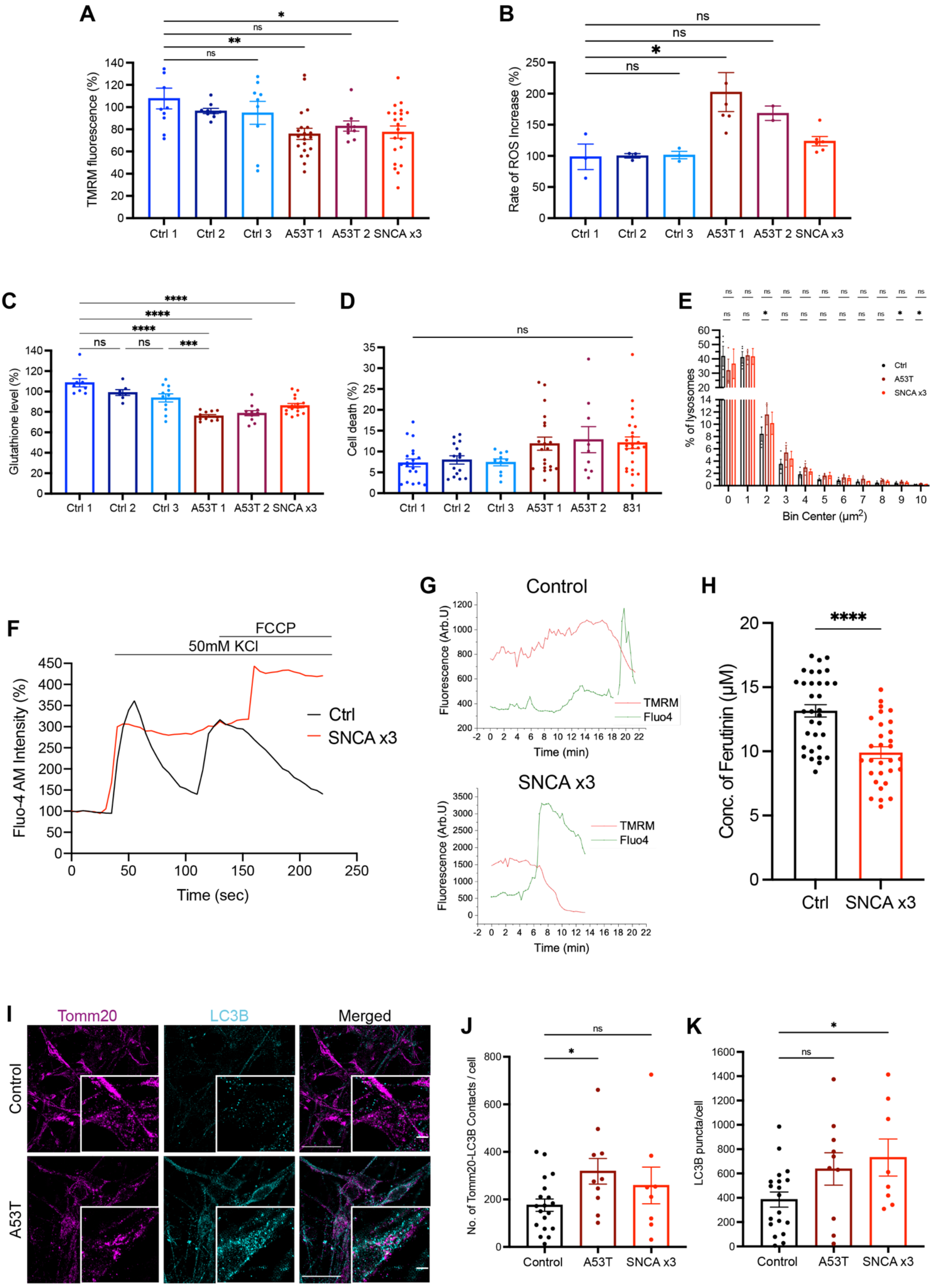

Supplementary Figure 8 – Late cellular pathology in mDA neurons.

(A) TMRM fluorescence plotted out for each line tested in >day 48 mDA neurons (ns $p > 0.05$, * $p < 0.05$, ** $p < 0.005$, one-way ANOVA). **(B)** Relative increase in ROS based on HEt ratiometric fluorescence plotted out for each line tested in >day 48 mDA neurons (ns $p > 0.05$, * $p < 0.05$, one-way ANOVA). **(C)** Relative percentage of endogenous glutathione levels based on MCB fluorescence plotted out for each line tested in >day 48 mDA neurons (ns $p > 0.05$, *** $p < 0.0005$, **** $p < 0.0001$, one-way ANOVA). **(D)** Basal cell death plotted out for each line tested in >day 48 mDA neurons (ns, $p > 0.05$, one-way ANOVA). **(E)** Histogram plot showing the percentage of total lysosomes in each set area bin (0-10μm^2) plotted out for each induction (ns $p > 0.05$, * $p < 0.05$, two-way ANOVA). **(F)** Representative single-cell trace showing Fluo-4 intensity in response to KCl and FCCP in day 62 mDA neurons. **(G)** Representative single-cell trace showing TMRM and Fluo-4 fluorescence as ferutinin is sequentially added to open mPTP in control and *SNCA* x3 day 62 neurons. **(H)** Quantification showing the concentration of ferutinin required for mPTP opening in control and *SNCA* x3 mDA neurons (Ctrl = 13.2 ±0.5, *SNCA* x3 = 9.9 ±0.5, **** $p < 0.0001$, Welch's t-test). **(I)** Structured illumination microscopy (SIM) images of control and A53T day 55 neurons probed for mitochondrial marker Tomm20, and the autophagosome marker LC3B. Scale bar = 20 μm). Smaller image depicts a zoom showing morphology and colocalization of the markers (scale bar = 2 μm). **(J)** Quantification of Tomm20/LC3B co-localisations in control, A53T, and *SNCA* x3 day 55 mDA neurons (Control = 176 ±26, A53T = 319 ±54, *SNCA* x3 = 259 ±77, ns $p > 0.05$, * $p < 0.05$, one-way ANOVA). **(K)** Quantification of LCB3 puncta per cell in control, A53T, and *SNCA* x3 neurons (Control = 385 ±62, A53T = 637 ±133, *SNCA* x3 = 731 ±152, ns $p > 0.05$, * $p < 0.05$, one-way ANOVA). All values plotted as ±SEM. All N numbers for each experiment can be found in supplementary table 5.

Supplementary Figure 9

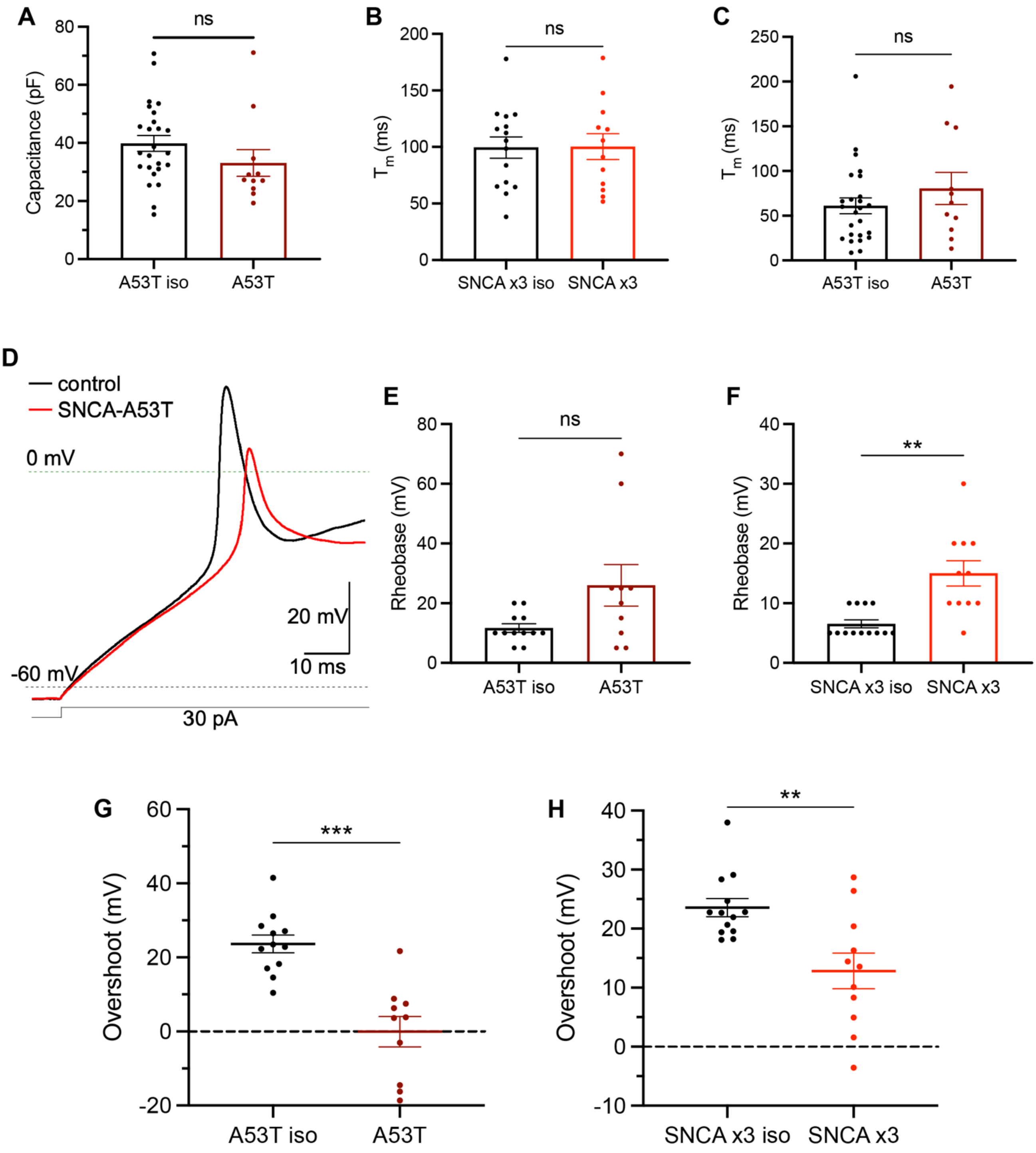

Supplementary Figure 9 – Electrophysiological pathology in late mDA neurons.

(A) Quantification showing the membrane capacitance in >day 70 A53T isogenic and A53T mDA neurons (ns, p >0.05, Welch's t-test). **(B)** Quantification showing the passive membrane property; time-constant in *SNCA* x3 isogenic and *SNCA* x3 mDA neurons (ns p >0.05, Welch's t-test). **(C)** Quantification showing the time-constant in A53T isogenic and A53T mDA neurons (ns, p >0.05). **(D)** Representative trace showing current induced AP form in control and A53T mDA neurons. **(E)** Quantification showing the AP rheobase in A53T isogenic and A53T mDA neurons (A53T isogenic = 11.7 ±1.4, A53T = 26.0 ±7.0, ns p > 0.05, Welch's t-test). **(F)** Quantification showing the AP rheobase in *SNCA* x3 isogenic and *SNCA* x3 mDA neurons (*SNCA* x3 isogenic = 6.5 ±0.67, *SNCA* x3 = 15.0 ±2.13, ** p = 0.0026, Welch's t-test). **(G)** Quantification of the AP overshoot in A53T isogenic and A53T mDA neurons (A53T isogenic = 23.6 ±2.4, A53T = -0.05 ±4.1, *** p = 0.0002, Welch's t-test). **(H)** Quantification of the AP overshoot in *SNCA* x3 isogenic and *SNCA* x3 mDA neurons (*SNCA* x3 isogenic = 23.6 ±1.5, *SNCA* x3 = 12.8 ±3.0, ** p = 0.0062, Welch's t-test). All values plotted as ±SEM. All N numbers for each experiment can be found in supplementary table 5.

Supplementary Table 1. hiPSC lines used in this study

hiPSC line	Mutation	Age of donor	Sex of donor	Source	RRID
No disease Control 1	None	78	Male	Reprogrammed in house, Kunath lab EDi046-A	
No disease Control 2	None	51	Male	Cedars Sinai iPSC Core Repository CS0002iCTR-nxx	
No disease Control 3	None	Unknown	Female	Thermo Fisher Scientific A18945	CVCL_RM92
No disease Control 4	None	64	Male	Coriell ND41866	CVCL_Y838
No disease Control 5	None	60-64	Female	EBiSC WTSli017-B HPSI0114i-lexy_1	CVCL_AE31
No disease Control 6	None	55-59	Male	EBiSC WTSli018-A HPSI0114i-kolf_3	CVCL_AE30
No disease Control 7	None	55-59	Male	EBiSC WTSli019-B HPSI0114i-iisa_1	CVCL_AE22
A53T 1 & isogenic control	*SNCA* A53T	54	Female	StemBANCC SFC828 (STBCi019-A)	CVCL_RB71
A53T 2	*SNCA* A53T	57	Male	StemBANCC SFC829 (STBCi023-C)	CVCL_RB78
SNCA x3	*SNCA* locus x3	Unknown	Female	StemBANCC SFC831 (STBCi024-C)	CVCL_RB81
SNCA x3 & isogenic control	*SNCA* locus x3	Unknown	Female	As reported [54] EDi001-B	CVCL_ZA47

Supplementary Table 2. List of antibodies used in this study.

Protein	Company	Catalogue	RRID	Species	Dilution
LMX1A	Abcam	ab139726	AB_2827684	Rabbit	1:500
FOXA2	Santa-Cruz Biotechnology	sc-374376	AB_10989742	Mouse	1:100
OTX2	R & D Systems	AF1979	AB_2157172	Goat	1:500
TH	Abcam	ab137869	AB_2801410	Rabbit	1:500 (ICC)<br>1:200 (Flow)
TH	Abcam	ab76442	AB_1524535	Chicken	1:500
TUJ1	Biolegend	801201	AB_2313773	Mouse	1:1000 (ICC)
Beta III Tubulin	Abcam	ab41489	AB_727049	Chicken	1:500 (ICC)<br>1:200 (Flow)
MAP2	Abcam	ab11267	AB_297885	Mouse	1:500
GIRK2	Alomone Labs	APC-006	AB_2040115	Rabbit	1:400
Total alpha-synuclein	Abcam	ab138501	AB_2537217	Rabbit	1:200
Filament alpha-synuclein	Abcam	ab209538	AB_2714215	Rabbit	1:50-1:100
Tomm20	Santa-Cruz	sc-17764	AB_628381	Mouse	1:1000
LC3B	Cell Signaling Technology	#3868	AB_2137707	Rabbit	1:300
LC3B	Abcam	ab48394	AB_881433	Rabbit	1:2000
Phosphorylated alpha-synuclein	Abcam	ab51253	AB_869973	Rabbit	1:1000
Goat pAb anti-Mouse IgG Alexa Fluor 488	Abcam	ab150113	AB_2576208	Goat	1:500
Goat pAb anti-Mouse IgG Alexa Fluor 555	Abcam	ab150114	AB_2687594	Goat	1:500
Goat pAb anti-Mouse IgG Alexa Fluor 647	Abcam	ab150115	AB_2687948	Goat	1:500
Goat pAb anti-Rabbit IgG Alexa Fluor 488	Abcam	ab150077	AB_2630356	Goat	1:500

Goat pAb anti-Rabbit IgG Alexa Fluor 555	Abcam	ab150078	AB_2722519	Goat	1:500
Goat pAb anti-Rabbit IgG Alexa Fluor 647	Abcam	ab150079	AB_2722623	Goat	1:500
Goat pAb anti-Chicken IgG Alexa Fluor 647	Abcam	ab150171	AB_2921318	Goat	1:500

Supplementary Table 3. List of TaqMan^{TM} Gene Expression probes used in this study.

Gene	Catalogue Number
FOXA2	Hs00232764_m1
LMX1A	Hs00898455_m1
EN1	Hs00154977_m1
TH	Hs00165941_m1
Nurr1	Hs00428691_m1
DAT	Hs00997374_m1
GIRK2	Hs01040524_m1
SNCA	Hs00240906_m1
GAPDH	Hs03929097_g1

Supplementary table 7: Protocols used in this study deposited on Protocols.io

Protocol	DOI
Human iPSC cell culture	dx.doi.org/10.17504/protocols.io.81wgby7dnvpk/v1
Generation of midbrain dopaminergic neurons	dx.doi.org/10.17504/protocols.io.x54v9j7ezg3e/v1
Single-cell RNA-seq	dx.doi.org/10.17504/protocols.io.6qpvr4dxpgmk/v1
Live-cell imaging: Calcium	dx.doi.org/10.17504/protocols.io.3byl4j7e8lo5/v1
Live-cell imaging: Mitochondria membrane potential	dx.doi.org/10.17504/protocols.io.e6nvwj997lmk/v1
Live-cell imaging: Cell death	dx.doi.org/10.17504/protocols.io.n2bvj8yywgk5/v1
Live-cell imaging: Reactive oxygen species (Superoxide)	dx.doi.org/10.17504/protocols.io.5qpvor55zv4o/v1
Fluorescent false neurotransmitter (FFN) DAT imaging	dx.doi.org/10.17504/protocols.io.36wgqj55xvk5/v1
Quantitative polymerase chain reaction (qPCR)	dx.doi.org/10.17504/protocols.io.36wgqj5rkvk5/v1
Immunocytochemistry	dx.doi.org/10.17504/protocols.io.q26g74w79gwz/v1
Sample preparation for single molecule localisation microscopy and iSIM and microscopy	dx.doi.org/10.17504/protocols.io.3byl4j77olo5/v1
Flow Cytometry	dx.doi.org/10.17504/protocols.io.kqdg3956eg25/v1
High Performance Liquid Chromatography (HPLC) and sample preparation	dx.doi.org/10.17504/protocols.io.yxmvm2ko5g3p/v1
Electrophysiology	dx.doi.org/10.17504/protocols.io.4r3l274mjg1y/v1
Proteomic sample preparation and analysis	dx.doi.org/10.17504/protocols.io.kxygxzrokv8j/v1
