# Supplementary material for: Protein aggregation and calcium dysregulation are hallmarks of familial Parkinson’s disease in midbrain dopaminergic neurons
Source: NPJ Parkinsons Dis. 2022 Nov 24;8:162. doi: 10.1038/s41531-022-00423-7 (PMC9691718; doi:10.1038/s41531-022-00423-7)
Supplement: Supplementary file 5 — Reporting Summary [file 41531_2022_423_MOESM5_ESM.pdf]

nature portfolio

Corresponding author(s): Sonia Gandhi

Last updated by author(s): Oct 20, 2022

Reporting Summary

Nature Portfolio wishes to improve the reproducibility of the work that we publish. This form provides structure for consistency and transparency in reporting. For further information on Nature Portfolio policies, see our Editorial Policies and the Editorial Policy Checklist.

Statistics

For all statistical analyses, confirm that the following items are present in the figure legend, table legend, main text, or Methods section.

n/a	Confirmed	
☐	☒	The exact sample size (*n*) for each experimental group/condition, given as a discrete number and unit of measurement
☐	☒	A statement on whether measurements were taken from distinct samples or whether the same sample was measured repeatedly
☐	☒	The statistical test(s) used AND whether they are one- or two-sided<br>*Only common tests should be described solely by name; describe more complex techniques in the Methods section.*
☒	☐	A description of all covariates tested
☐	☒	A description of any assumptions or corrections, such as tests of normality and adjustment for multiple comparisons
☐	☒	A full description of the statistical parameters including central tendency (e.g. means) or other basic estimates (e.g. regression coefficient) AND variation (e.g. standard deviation) or associated estimates of uncertainty (e.g. confidence intervals)
☐	☒	For null hypothesis testing, the test statistic (e.g. *F*, *t*, *r*) with confidence intervals, effect sizes, degrees of freedom and *P* value noted<br>*Give P values as exact values whenever suitable.*
☒	☐	For Bayesian analysis, information on the choice of priors and Markov chain Monte Carlo settings
☒	☐	For hierarchical and complex designs, identification of the appropriate level for tests and full reporting of outcomes
☒	☐	Estimates of effect sizes (e.g. Cohen's *d*, Pearson's *r*), indicating how they were calculated

Our web collection on statistics for biologists contains articles on many of the points above.

Software and code

Policy information about availability of computer code

Data collection	LSM 710, 880 Confocal microscope (Zeiss), Andor iQ 3, Elyra PS.1 (Zeiss), Oxford Nanoimager (Oxford Nanoimaging LTD), Olympus IX83 inverted microscope, Olympus BX51WI upright microscope, LSRii (BD) cell sorter, QuantSudio 6 Flex Real-Time PCR System (Applied Biosystems).
Data analysis	FIJI-win64, Cell Ranger v3.0.2, Seurat v3.1.0, R-3.6.1 (R Core Team, 2019), scVelo v0.2.2, Biognosys Spectronaut pulsar (version: 14.0), Biognosys Spectronaut pulsar software suite (version: 16.0.220606.5300), Perseus software suite, EZChrom Elite™ chromatography data system software, version 3.1.7, Python 3.8, sklearn v0.24.2, Micro-Manager v2.0, FlowJo,<br>The code used for analysis has been deposited online:<br>(https://github.com/strohstern/Transcriptomic_signatures_iPSC_derived_dopamine_neurons), https://doi.org/10.5281/zenodo.7123756

For manuscripts utilizing custom algorithms or software that are central to the research but not yet described in published literature, software must be made available to editors and reviewers. We strongly encourage code deposition in a community repository (e.g. GitHub). See the Nature Portfolio guidelines for submitting code & software for further information.

Data

Policy information about availability of data

All manuscripts must include a data availability statement. This statement should provide the following information, where applicable:

- Accession codes, unique identifiers, or web links for publicly available datasets
- A description of any restrictions on data availability
- For clinical datasets or third party data, please ensure that the statement adheres to our policy

All data is deposited on repositories available in Zenodo: DOI https://doi.org/10.5281/zenodo.7138359.
Single-cell RNA-seq raw data were deposited to NCBI Gene Expression Omnibus. The accession code for the data is: GSE213569 (https://www.ncbi.nlm.nih.gov/geo/).
Mass spectrometry proteomic raw data and search engine output files were deposited to PRIDE ProteomeExchange repository and the data can be accessed using the identifier: PXD035500. The login details are, Username: reviewer_pxd035500@ebi.ac.uk. Password: 6YuJLsE7.

Human research participants

Policy information about studies involving human research participants and Sex and Gender in Research.

Reporting on sex and gender	*Use the terms sex (biological attribute) and gender (shaped by social and cultural circumstances) carefully in order to avoid confusing both terms. Indicate if findings apply to only one sex or gender; describe whether sex and gender were considered in study design whether sex and/or gender was determined based on self-reporting or assigned and methods used. Provide in the source data disaggregated sex and gender data where this information has been collected, and consent has been obtained for sharing of individual-level data; provide overall numbers in this Reporting Summary. Please state if this information has not been collected. Report sex- and gender-based analyses where performed, justify reasons for lack of sex- and gender-based analysis.*
Population characteristics	*Describe the covariate-relevant population characteristics of the human research participants (e.g. age, genotypic information, past and current diagnosis and treatment categories). If you filled out the behavioural & social sciences study design questions and have nothing to add here, write "See above."*
Recruitment	*Describe how participants were recruited. Outline any potential self-selection bias or other biases that may be present and how these are likely to impact results.*
Ethics oversight	*Identify the organization(s) that approved the study protocol.*

Note that full information on the approval of the study protocol must also be provided in the manuscript.

Field-specific reporting

Please select the one below that is the best fit for your research. If you are not sure, read the appropriate sections before making your selection.

☒ Life sciences ☐ Behavioural & social sciences ☐ Ecological, evolutionary & environmental sciences

For a reference copy of the document with all sections, see nature.com/documents/nr-reporting-summary-flat.pdf

Life sciences study design

All studies must disclose on these points even when the disclosure is negative.

Sample size	The sizes of the sample for each experiment was selected to ensure that the technical (number of cells, numbers of fields of view, and number of coverslips), and biological (number of hiPSC lines, and number of neuronal inductions) variation was adequately captured. The size of the samples were not predetermined, but are similar to others reported in the literature.
Data exclusions	No data were excluded.
Replication	We repeated experiments in order to account for technical and biological variability, using at least 2 independent neuronal inductions.
Randomization	Numbers for cell lines were randomly allocated for each cell plating, and the order at which experiments was performed on the samples was randomized for each experiment.
Blinding	When blinding is not possible, data were collected and analyzed without bias, and normalized to several different biological replicates.

Reporting for specific materials, systems and methods

We require information from authors about some types of materials, experimental systems and methods used in many studies. Here, indicate whether each material, system or method listed is relevant to your study. If you are not sure if a list item applies to your research, read the appropriate section before selecting a response.

Materials & experimental systems

n/a	Involved in the study
☐	☒ Antibodies
☐	☒ Eukaryotic cell lines
☒	☐ Palaeontology and archaeology
☒	☐ Animals and other organisms
☒	☐ Clinical data
☒	☐ Dual use research of concern

Methods

n/a	Involved in the study
☒	☐ ChIP-seq
☐	☒ Flow cytometry
☒	☐ MRI-based neuroimaging

Antibodies

Antibodies used	All antibodies used have RRIDs, which are listed below: LMX1A (ab139726 AB_2827684), FOXA2 (sc-374376 AB_10989742), OTX2 (AF1979 AB_2157172), TH (ab137869 AB_2801410), TH (ab76442 AB_1524535), TUJ1 (801201 AB_2313773), Beta III Tubulin (ab41489 AB_727049), MAP2 (ab11267 AB_297885), GIRK2 (APC-006 AB_2040115), Total alpha-synuclein (ab138501 AB_2537217), Filament alpha-synuclein (ab209538 AB_2714215), Tomm20 (sc-17764 AB_628381), LC3B (#3868 AB_2137707), LC3B (ab48394 AB_881433), Phosphorylated alpha-synuclein (ab51253 AB_869973), Goat pAb anti-Mouse IgG Alexa Fluor 488 (ab150113 AB_2576208), Goat pAb anti- Mouse IgG Alexa Fluor 555 (ab150114 AB_2687594), Goat pAb anti- Mouse IgG Alexa Fluor 647 (ab150115 AB_2687948), Goat pAb anti- Rabbit IgG Alexa Fluor 488 (ab150077 AB_2630356)
Validation	All commercial primary antibodies were validated by the manufacturer, Abcam (https://www.abcam.com/), Santa Cruz Biotechnology (https://www.scbt.com/home), R&D Systems (https://www.rndsystems.com/products/antibodies), Alomone labs (https://www.alomone.com/about-alomone-labs/antibodies), Cell Signaling Technology (https://www.cellsignal.co.uk/), BioLegend (https://www.biolegend.com/en-gb)

Eukaryotic cell lines

Policy information about cell lines and Sex and Gender in Research

Cell line source(s)	Human induced pluripotent stem cells: fibroblast reprogrammed.
Authentication	None of the cell lines used were authenticated.
Mycoplasma contamination	All hiPSC lines tested were mycoplasma negative.
Commonly misidentified lines (See ICLAC register)	None of the cell lines are commonly misidentified lines.

Flow Cytometry

Plots

Confirm that:

☒ The axis labels state the marker and fluorochrome used (e.g. CD4-FITC).

☒ The axis scales are clearly visible. Include numbers along axes only for bottom left plot of group (a 'group' is an analysis of identical markers).

☒ All plots are contour plots with outliers or pseudocolor plots.

☒ A numerical value for number of cells or percentage (with statistics) is provided.

Methodology

Sample preparation	hiPSC-derived midbrain dopaminergic neurons. Neurons were harvested in a single-cell suspension, fixed with PFA and immunolabelled for relevant markers. Full protocol is described in the manuscript.
Instrument	LSR ii (BD) flow cytometer

Software

FlowJo

Cell population abundance

10000 cells events per sample

Gating strategy

The single-cell population was then gated to include DAPI positive only cells (negative control). The gating threshold for measured channels was determined using the control lacking the antibody of interest (Fluorescence minus one (FMO) control), for both channels being recorded.

☒ Tick this box to confirm that a figure exemplifying the gating strategy is provided in the Supplementary Information.
